# Co_3_O_4_/CeO_2_ and Co_3_O_4_/ZrO_2_ Composites as Heterogeneous Catalysts for Selective Aerobic Oxidation of Vanillyl Alcohol

**DOI:** 10.1002/open.70228

**Published:** 2026-05-14

**Authors:** Hashini T. Abeyrathna, Chamodi L. Fernando Thibiripalage, Huai Yong Zhu, Yichao Jin, Aaron Micallef, Eric R. Waclawik

**Affiliations:** ^1^ School of Chemistry and Physics Queensland University of Technology Brisbane Australia; ^2^ Central Analytical Research Facility (CARF) Queensland University of Technology Brisbane Australia

**Keywords:** biomass valorisation, heterogeneous catalysis, metal oxide composites, nanomaterial characterization, selective oxidation

## Abstract

There are significant benefits in achieving efficient, selective conversion of biomass‐derived substrates into value‐added chemicals. This study focuses on binary heterogeneous catalysts composed of Co_3_O_4_ supported on CeO_2_ and ZrO_2_, which were highly active for the selective oxidation of vanillyl alcohol to vanillin under base‐free conditions. Co_3_O_4_/CeO_2_ and Co_3_O_4_/ZrO_2_ composites were synthesised using a facile coprecipitation method followed by calcination to activate the catalyst materials. Chemical and structural properties of these systems were characterized with X‐ray diffraction, X‐ray photoelectron spectroscopy, scanning electron microscopy, transmission electron microscopy, energy‐dispersive X‐ray spectroscopy, and N_2_ sorption measurements to establish the structure–activity relationship. Cyclic thermal gravimetric analysis experiments quantified the oxygen storage capacity of the catalysts, establishing oxygen buffering capacity and faster lattice oxygen exchange as key factors that govern catalytic turnover. The activity of Co_3_O_4_/CeO_2_ for vanillyl alcohol oxidation was found to be superior compared to Co_3_O_4_/ZrO_2_ catalyst under different temperatures and reaction times at atmospheric pressure. Enhanced activity of Co_3_O_4_/CeO_2_ was explored using quenching studies, in situ DRIFTS and electron paramagnetic resonance experiments. These studies established the key role of superoxide (O_2_^•−^) in the oxidation reaction pathway, while hydroxyl radicals (HO^•^) made a negligible contribution. A plausible catalytic pathway for the selective oxidation of vanillyl alcohol with Co_3_O_4_/CeO_2_ catalyst was based on these results.

## Graphical abstract

This work compares binary metal oxides of Co_3_O_4_/CeO_2_ and Co_3_O_4_/ZrO_2_ to elucidate the structure–activity relationship on the selective oxidation of vanillyl alcohol to vanillin, highlighting the Co_3_O_4_/CeO_2_ system that enables high efficiency and selectivity under mild, green reaction conditions.

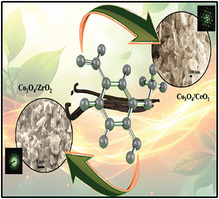

## 1 Introduction

Lignin is the most abundant renewable aromatic polymer in the ecosphere, accounting for more than 20% of lignocellulosic biomass [1, 2]. Due to the complex heterogeneous structure and lack of efficient depolymerisation and modification steps, more than 98% of lignin is combusted as a source of energy rather than being converted into high‐value commercial products [3, 4, 5]. Lignin depolymerization produces a range of functionalized phenolic molecules such as *p*‐coumaryl, *p*‐sinapyl, vanillyl, and veratryl alcohols, etc., which can serve as valuable intermediates for fuels, polymers, and fine chemicals [6]. Among these phenolic compounds, vanillyl alcohol (4‐hydroxy‐3‐methoxybenzyl alcohol) holds significance due to its direct link to vanillin (4‐hydroxy‐3‐methoxybenzaldehyde), one of the most important value‐added chemicals derived from lignin [5]. Vanillin is extensively employed in the food, fragrance, cosmetics, and pharmaceutical industries, with global demand increasing steadily each year [6, 7, 8].

The selective oxidation of vanillyl alcohol to produce functionalized aromatic chemicals has been extensively studied during the past decade [5, 7, 9]. Conventional processes to achieve this conversion often rely on stoichiometric oxidants or homogeneous metal complexes (based on Fe, Co, V, Mn, Au, or Re), which are directly associated with issues of poor recyclability, multistep synthesis, costly separation, and generation of secondary waste streams [10, 11]. Furthermore, achieving both high conversion of vanillyl alcohol and selectivity toward vanillin under mild, base‐free conditions remains nontrivial due to competing side reactions, which lead to over‐oxidised products [6, 12]. The development of robust, heterogeneous catalysts that are highly active, selective, recyclable, and that overcome existing limitations, has become an area of intense research interest.

Cobalt is an attractive element for oxidation reactions due to its abundance, its low cost relative to the noble metals, which can also be used to achieve this task, cobalt's versatile redox chemistry, and its strong ability to activate molecular oxygen [10, 13]. The Co_3_O_4_ spinel is the thermodynamically stable form of cobalt oxide under ambient conditions, with a lattice structure that consists of one tetrahedrally coordinated Co^2+^ site and two octahedrally coordinated Co^3+^ sites [14, 15, 16]. Their oxidation states and reactivity are strongly influenced by the local geometric and electronic structures of the cobalt ion [14]. Co^2+^ and Co^3+^ are considered thermodynamically competitive under typical ambient conditions, and the redox mechanisms that connect the two states are responsible for their success in partial oxidation catalysis. Co^3+^ on the Co_3_O_4_ catalyst surface is thought to provide the active sites for oxidation, while Co^2+^ is considered inactive [17]. Pure Co_3_O_4_ does have some inherent limitations, however, including limited surface area, risk of metal leaching, and moderate selectivity [18]. Therefore, the formation of binary oxide systems with compatible supports has been popularised to enhance the catalytic activity and stabilise the active sites while inhibiting metal ion leaching [18].

In the present study, ZrO_2_ and CeO_2_ have been explored as two promising supports for cobalt‐based binary heterogeneous oxidation catalysis. ZrO_2_ has three polymorphic phases, namely tetragonal, monoclinic, and cubic; it is considered a nontoxic, reducible metal oxide containing both acidic and basic sites, which makes it a versatile support for oxidation catalysis [19, 20, 21]. Moreover, ZrO_2_ forms strong interactions with other metal components, thus it can promote the spillover of reaction intermediates between a metal and the support, which is extremely important to the migration of the active species between two catalytic surfaces [22]. In contrast, CeO_2_ is unique among n‐type semiconductors because of its remarkable oxygen storage and release capacity and oxygen mobility [23, 24]. The Ce^3+^ and Ce^4+^ redox couple mobilises lattice oxygen for catalytic reactions and regenerates active oxygen species by facilitating oxidation reactions [18, 25, 26, 27]. Recent advances in cerium‐based systems have further demonstrated that tuning oxygen vacancies, surface defects, and metal support interactions can significantly enhance their applicability in different fields, including oxidative catalysis [28, 29, 30, 31]. These features enable CeO_2_‐supported systems to promote oxygen vacancy formation and improve the charge transfer between catalytic components [18].

Though both ZrO_2_ and CeO_2_ offer several advantages as catalyst supports, direct comparisons of these nonreducible and reducible oxides with Co_3_O_4_ as heterogeneous catalysts for vanillyl alcohol oxidation are scarce. Numerous studies have recently focused either on noble metal‐based catalysts (such as Au, Pd, and Cu supported on oxides) that are likely to be economically unsustainable or on organometallic complexes that are inefficient in recyclability [6, 24, 32, 33]. Meanwhile, a better understanding of how different supports, such as ZrO_2_ and CeO_2_, influence cobalt's redox behaviour, active site stabilization, and catalytic activity for the selective oxidation reactions is still lacking. Addressing this knowledge gap is essential for designing cost‐effective and sustainable catalysts for lignin valorisation. The present study explores the activity of Co_3_O_4_/CeO_2_ and Co_3_O_4_/ZrO_2_ composites for the selective oxidation of vanillyl alcohol under base‐free conditions. To place the performance of the present Co_3_O_4_‐based catalysts in context, Table 1 compares them with representative noble metal and nonprecious metal systems reported for the selective oxidation of vanillyl alcohol. Compared to the existing literature, this study demonstrated complete conversion of near‐quantitative selectivity for vanillyl alcohol oxidation under exceptionally mild, base‐free and low‐oxygen conditions with Co_3_O_4_/CeO_2_ catalyst. Furthermore, in situ electron paramagnetic resonance (EPR) and in situ DRIFTS provided a mechanistic insight. A comprehensive deactivation assessment confirms that activity loss is primarily due to structural deterioration, coke deposition and surface hydroxyl accumulation rather than metal leaching.

**TABLE 1:** Different heterogeneous catalysts and their activity and reaction conditions for the selective oxidation of vanillyl alcohol.

Catalyst	Reaction conditions	Conversion%	Selectivity%	Reference
Mn–Co mixed oxide	Aerobic, base‐free, 21 bar pressure, 140°C	64	74	[34]
LaFeO_3_	O_2_, base‐free, 1 MPa, 180°C	100	33	[35]
Cu(II) complexes	Air, 70°C, with TEMPO	100	100	[6]
Co_3_O_4_ nanoparticles	Air, high pressure, 80°C	80	98	[36]
CeO_2_‐Co_3_O_4_ (9:1 molar ratio)	Atmospheric O_2_, 140°C	∼100	∼100	[24]
Ag‐Pd/ZrO_2_	O_2_, base‐free, 3 bar, 120°C	100	95	[37]
Cu‐Ce oxides	O_2_, 130°C, base‐free	95	100	[38]
Ce_0.8_Zr_0.2_O_2_	O_2_, base‐free, 20 bar, 140°C	∼98	∼99	[39]
Cu–Mn mixed oxide	H_2_O_2_, NaOH, 85°C & aerobic conditions, 21 bar, 120°C	94	99	[40]
Ce_0.8_Fe_0.2_O_2_	O_2_, 20 bar, 140°C	91	99	[41]

This comparison study of Co_3_O_4_/CeO_2_ and Co_3_O_4_/ZrO_2_ heterogeneous catalysts for the selective oxidation of vanillyl alcohol aims to deliver new insights into the rational design of efficient and sustainable heterogeneous catalysts.

## 2 Experimental

### 2.1 Materials

All chemicals were of analytical grade and used as received without further purification. Cerium (III) acetate hydrate (≥99.9%), zirconium (IV) chloride (≥99.5%), cobalt (II) nitrate hexahydrate (98%), sodium hydroxide (≥99%), 4‐hydroxy‐3‐methoxybenzyl alcohol (98%), acetonitrile (HPLC grade, ≥99.9%), *N*‐*tert*‐butyl‐α‐phenylnitrone (PBN, ≥98%), and 5,5‐dimethyl‐1‐pyrroline *N*‐oxide (DMPO, ≥98.0%) were acquired from Merck Life Science Pty Ltd. and used as received.

### 2.2 Co_3_O_4_/CeO_2_ (1:1) Composite Preparation

1.5 g of Ce(OAc)_3_·xH_2_O was added to 50 mL of deionised water and stirred magnetically until a well‐dispersed suspension formed. 1.5 g of Co(NO_3_)_2_·6H_2_O was then added to the suspension and stirred magnetically at 450 rpm for 30 min. 100 mL of 0.3 M NaOH was added dropwise to the suspension, and the mixture was left undisturbed for 12 h. The solid component of the resulting suspension was collected via centrifugation and washed several times with RO water and ethanol. The washed precipitate was then dried in an oven at 60°C overnight, and then the dried powder was calcined at 500°C for 2 h. The ratio between the Co and CeO_2_ was calculated to be 1:1 based on the precursor wt%. Co_3_O_4_/CeO_2_ 1:2 and 2:1 weight ratio catalysts were also synthesised by varying the initial precursor masses accordingly.

### 2.3 Co_3_O_4_/ZrO_2_ (1:1) Composite Preparation

1.5 g of ZrCl_4_ was added to 50 mL of deionized water and magnetically stirred until a well‐dispersed suspension formed. 1.5 g of Co(NO_3_)_2_·6H_2_O was then added to the suspension and stirred magnetically at 450 rpm for 30 min. 100 mL of 0.3 M NaOH was then added dropwise to the suspension, and the mixture was left undisturbed overnight. Solids in the resulting suspension were then collected via centrifugation and washed several times with RO water and ethanol. The obtained precipitate was dried in an oven at 60°C overnight, and the dried powder was then calcined at 500°C for 2 h. The ratio between the Co and ZrO_2_ was calculated to be 1:1 based on the precursor wt%.

### 2.4 Characterization Methods

Powder X‐ray diffraction (XRD) was used to characterise the phase of all catalyst materials using a Bruker D8 Advanced Co/Cr XRD operating with CoKα1 radiation (λ = 1.78897 Å) in Bragg–Brentano geometry. A Kratos AXIS Supra photoelectron spectrometer was used to analyse the X‐ray photoelectron spectroscopy (XPS) spectra of the samples with 165 mm under UHV conditions. Monochromatic Al Kα radiation (1486.6 eV) at 225 W (15 kV, 15 mA) was the incident radiation source. Binding energies were calibrated by adding contaminant carbon (C 1*s* = 284.8 eV) and peaks were deconvoluted using Shirley fitting in CASA XPS software. The morphology and composition of the catalysts were analysed with a JEOL 700F Scanning electron microscope and energy‐dispersive X‐ray spectroscopy mapping. Raman spectra were obtained from the Qontor Raman Spectrometer in the range of 100–800 nm. Nitrogen sorption measurements were taken with the Micromeritics 3 Flex surface area and porosity system on degassed samples treated under the Micromeritics 3 Flex Smart VacPrep degas system. High‐resolution transmission electron microscopy (TEM) images of the samples were obtained from a JEOL TEM‐2100 instrument with an acceleration voltage of 200 kV. Oxygen storage capacity (OSC) measurements were carried out on Netzsch STA 449F3 Jupiter Simultaneous Thermal Analyser, alternatively introducing O_2_ and reductive H_2_ gas to the system. Fourier transform infrared spectroscopy (FTIR) spectra were measured on a Nicolet iS50 spectrometer, incorporating a single‐bounce diamond attenuated total reflectance (ATR) module with a dedicated MCT detector (Thermo Fisher Scientific Inc., USA). In situ FTIR studies of the oxidation of vanillyl alcohol on the catalyst were conducted at 70°C under atmospheric air by using a commercial in situ DRIFTS cell (HVC‐DRM‐5, Harrick's Scientific) possessing ZnSe windows and equipped with a resistive heating device. A drop of 1 mmol vanillyl alcohol in acetonitrile was added to the catalyst bed in the DRIFTS cell, and data were collected every 5 min at 24 scans for 2 h, in the spectral range of 800–4000 cm^−1^. EPR spectroscopy was performed on a Bruker Magnettech ESR5000 continuous‐wave benchtop spectrometer operating at X‐band. Spectra were recorded at room temperature in quartz tubes. Simulation and fitting of experimental spectra were performed using the SpinFit Liquids module in the spectrometer's ESRStudio software package. EPR spectroscopy spin trapping experiments were conducted with DMPO and PBN as radical trapping agents to explore the formation of HO^•^ and O_2_^•−^ radicals. Generally, in situ EPR experiments were carried out using a 3 mm (outer diameter) quartz tube filled with 1 mg of catalyst dispersed in a 150 μL solution of the radical trapping agent (0.5 mM), while maintaining the sample temperature at 70°C.

### 2.5 Procedure for the Oxidation of Vanillyl Alcohol

10 mL Pyrex glass tubes were charged with 20 mg of the catalyst and 3 mL of 3 mM vanillyl alcohol in acetonitrile. The reaction mixture was purged with oxygen for 5 min before the reaction. The tubes were completely sealed, and the reaction was run at 1 atm pressure and a constant temperature, maintained with an oil bath. For analysis, 1 mL aliquots of the reaction mixture were collected at predetermined intervals and filtered through a Millipore filter (pore size 0.45 μm) to remove the catalyst particles. High‐performance liquid chromatography (Dionex Ultimate HPLC) and an LTQ Orbitrap Elite mass spectrometer were used to analyse the product distribution of the filtrates.

## 3 Results and Discussion

### 3.1 Catalyst Characterisation

**FIGURE 1:**
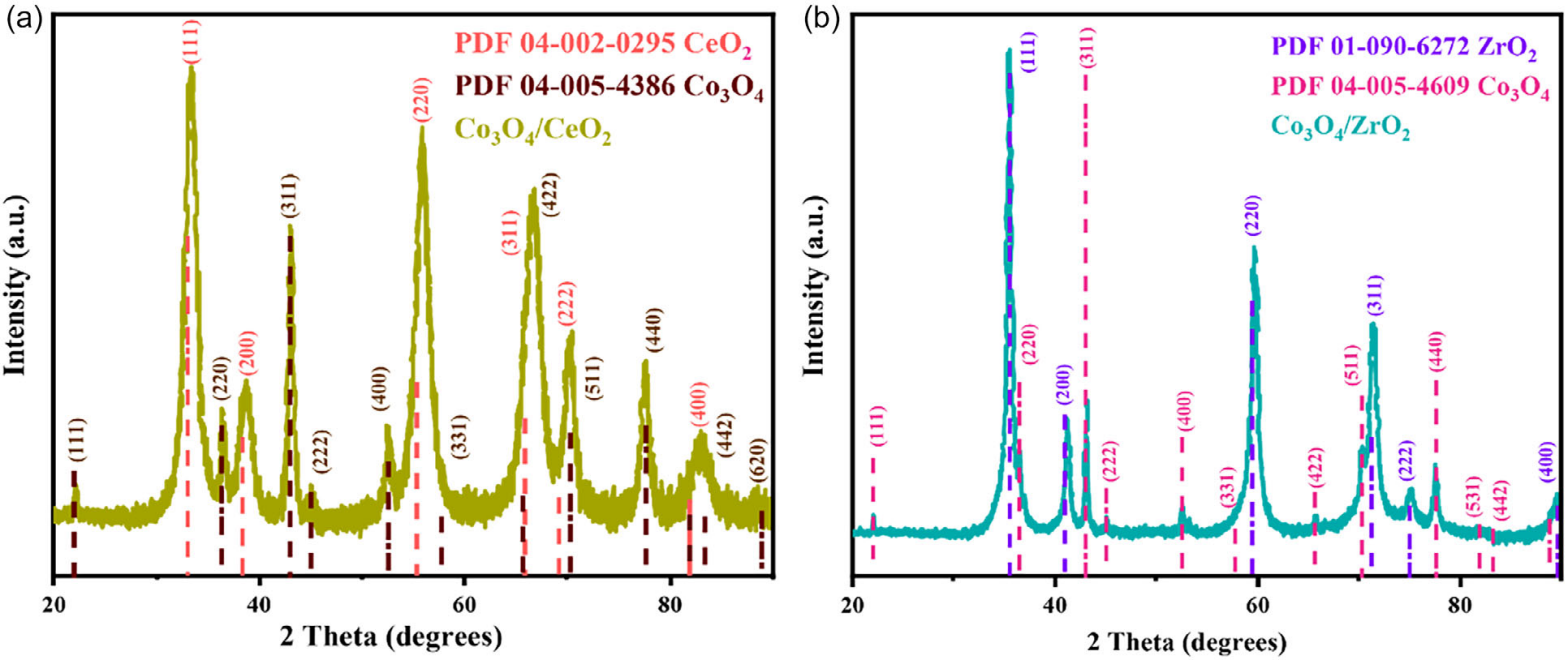
Powder XRD pattern of the (a) Co_3_O_4_/CeO_2_ catalyst and (b) Co_3_O_4_/ZrO_2_ catalyst.

Synthesised Co_3_O_4_/CeO_2_ and Co_3_O_4_/ZrO_2_ composite materials were characterised by XRD to identify their crystal phases and establish how the pristine CeO_2_ and ZrO_2_ crystal structures changed upon doping of cobalt into the support. The XRD patterns of the Co_3_O_4_/CeO_2_ catalyst (Figure 1a) matched well with the CeO_2_ (PDF 04‐002−0295) and Co_3_O_4_ (PDF 04‐005−4386), confirming the presence of discrete CeO_2_ and Co_3_O_4_ phases only, with no additional peaks corresponding to mixed cobalt‐ceria oxide phases or unintended secondary phases.

XRD patterns of Co_3_O_4_/CeO_2_ and Co_3_O_4_/ZrO_2_ catalysts were analysed using the Rietveld refinement method embedded in the Bruker DIFFRAC TOPAS software (version 7) to define the lattice parameters and the crystallite size of the materials. In the Co_3_O_4_/CeO_2_ system, average crystallite sizes for CeO_2_ and Co_3_O_4_ were 5.2 and 13.0 nm, respectively. Shifts in the positions of the XRD peaks of Co_3_O_4_/CeO_2_ reflect the changes in the lattice parameters of the unit cells. For CeO_2_, the unit cell size decreased from 5.4555 to 5.4277 Å, and for Co_3_O_4_, it increased from 8.0835 to 8.113 Å. These changes can occur due to the formation of solid solutions or changes in the Ce redox state [42]. The reduction in the lattice constant of ceria occurs as Co^3+^ ions (*r* = 0.61 Å) are partially incorporated into the CeO_2_ lattice, replacing larger Ce^3+^ ions (*r* = 1.01 Å), and is indicative of the formation of a Co–Ce–O solid solution [42, 43]. The observed expansion of the Co_3_O_4_ lattice parameter can be attributed to the incorporation of larger Ce^3+^ ions into the Co_3_O_4_ crystal structure [42, 43].

**FIGURE 2:**
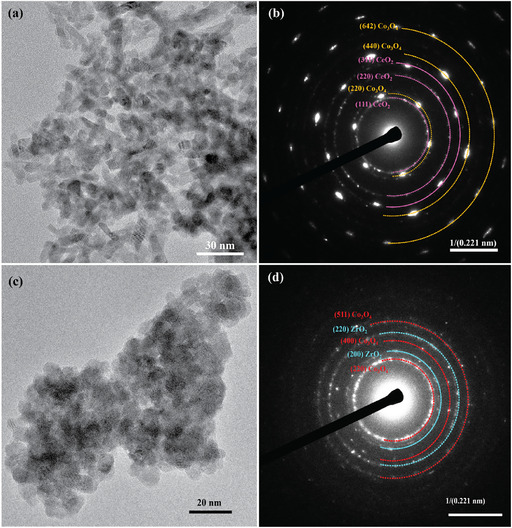
(a) TEM image of Co_3_O_4_/CeO_2_, (b) SAED pattern of Co_3_O_4_/CeO_2_, (c) TEM image of Co_3_O_4_/ZrO_2_, and (d) SAED pattern of Co_3_O_4_/ZrO_2_.

The addition of cobalt ions in the precursor solution of ZrCl_4_ forms cubic zirconia (c‐ZrO_2_) nanopowder through a simple low‐temperature reaction, followed by calcination. As depicted in Figure 1b, XRD analysis of the cobalt‐doped ZrO_2_ samples gave an XRD pattern corresponding to the ZrO_2_ cubic phase (space group of Fm‐3m‐225), where peak positions matched with the standard file PDF‐01‐090‐6272. Similar stabilization of the high‐temperature cubic phase of ZrO_2_ has previously been achieved only through synthesis at ambient temperatures by doping zirconia with yttrium and with calcium [44]. Cobalt doping produced additional peaks that were identified as the cubic Co_3_O_4_ phase (PDF‐04‐005‐4609). As depicted by the Scanning electron microscopy (SEM) and SEM‐EDS analysis (Figure 2), Co_3_O_4_ nanoparticles were dispersed on the surface of the ZrO_2_ substrate without lattice deformation of bare Co_3_O_4_. Generally, the radius of Zr^4+^ (72 pm) is slightly larger than that of Co^3+^ (65 pm) and smaller than the radius of Co^2+^ (78 pm) [45]. This allows Co^3+^ to incorporate into tetragonal ZrO_2_, changing the active phase of the material to the cubic phase upon addition of cobalt (Figure S1). This could be further confirmed by the changes in the lattice parameters of the bare ZrO_2_ and Co_3_O_4_/ZrO_2_. The lattice parameter of bare ZrO_2_ increases from 5.0942 to 5.1076 Åin the composite, while that for cubic Co_3_O_4_ increases to 8.400 Åin the composite relative to 8.072 Åin the bare Co_3_O_4_.

Morphology and structural analysis of the two composites were performed using TEM and SEM techniques. As shown in the HR‐TEM image of the Co_3_O_4_/CeO_2_ catalyst (Figure 2a), the catalyst formed with a randomly oriented, elongated morphology. The high crystallinity of the material is evident from the clear lattice fringes observed in the HR‐TEM images. The successful formation of the composite is further supported by the selected area electron diffraction (SAED) pattern, which displays distinct diffraction rings corresponding to the crystal planes of both CeO_2_ and Co_3_O_4_. Lattice spacings of 3.15, 1.93, and 1.64 Åwere indexed to the (111), (220), and (311) planes of CeO_2_, respectively. Similarly, diffraction rings with spacings of 2.86, 1.43 and 1.08 Åwere attributed to the (220), (440), and (642) planes of Co_3_O_4_. These results confirm the successful integration of both crystalline phases in the composite structure.

Rather than being elongated, TEM analysis of the Co_3_O_4_/ZrO_2_ catalyst identified that irregularly shaped particles were produced (Figure 2c). The HR‐TEM images display well‐defined lattice fringes, indicating the crystalline nature of the sample. SAED analysis further confirms the coexistence of both Co_3_O_4_ and ZrO_2_ phases. Lattice spacings of 2.89, 2.07, and 1.55 Å were indexed to the (220), (400), and (511) planes of Co_3_O_4_, respectively. Additionally, the ZrO_2_ phase exhibited lattice spacings of 2.50 and 1.83 Å, corresponding to the (200) and (220) planes.

**FIGURE 3:**
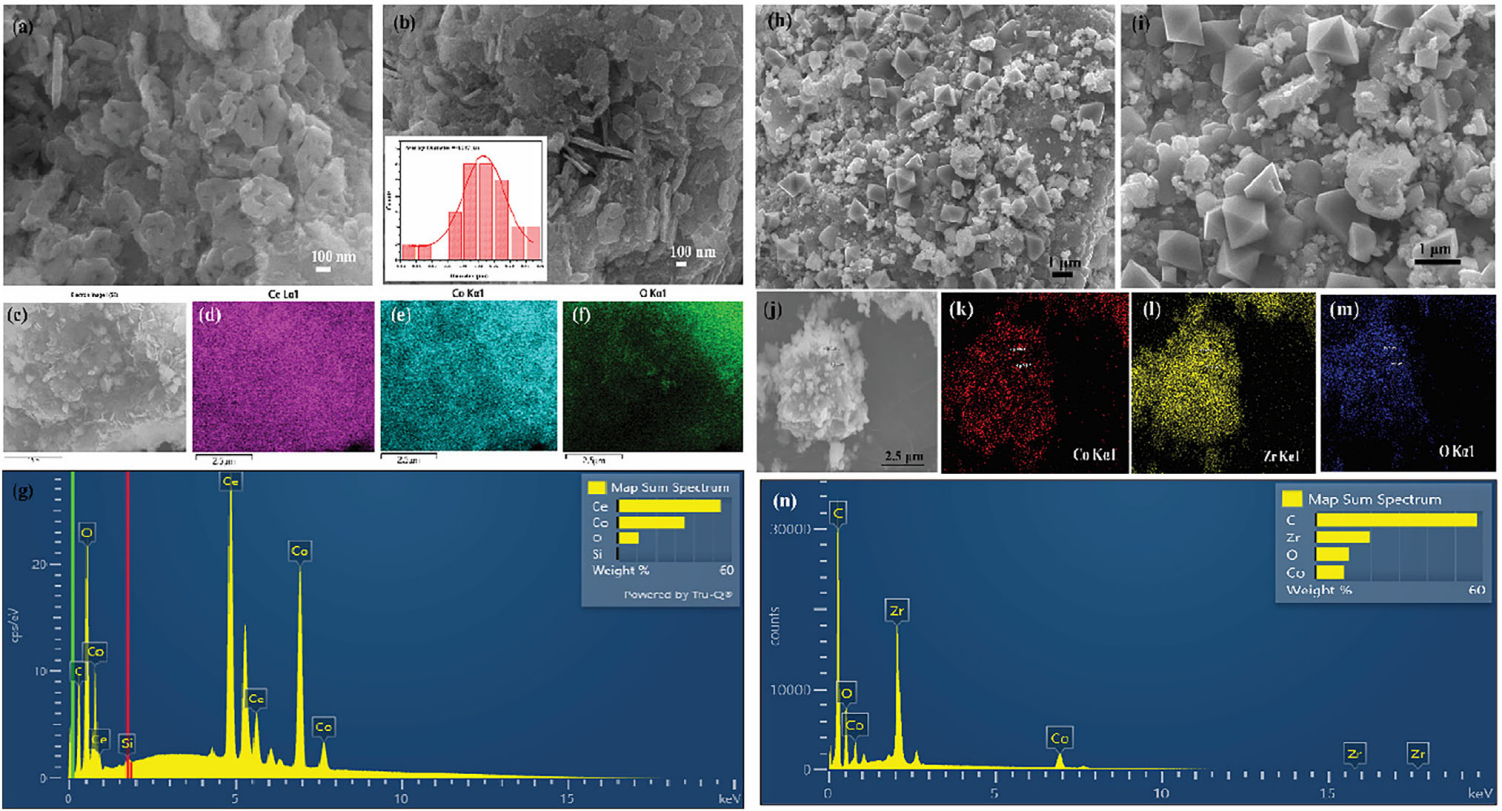
(a,b) SEM images of Co_3_O_4_/CeO_2_ catalysts with average particle diameter distribution, (c) electron image of Co_3_O_4_/CeO_2_ selected for EDS analysis, (d) EDS map of Ce wt%, (e) EDS map of Co wt%, (f) EDS map of O wt%, (g) EDS point ID analysis (h, i) SEM images of Co_3_O_4_/ZrO_2_, (j) electron image of Co_3_O_4_/ZrO_2_ used for EDS analysis, (k) EDS map of Co wt%, (l) EDS map of Zr wt%, (m) EDS map of O wt%, and (n) EDS point ID analysis.

The surface morphology of the two composite materials was examined by SEM analysis. As shown in Figure 3a,b), the Co_3_O_4_/CeO_2_ sample formed with an irregular plate‐like morphology with visible voids or holes. The plates have an average diameter of 0.247 μm, as determined by ImageJ software. The presence of the holes and loosely packed plate structures indicates that the particles aggregate in a way that forms porous architecture, potentially contributing to a higher surface area, expected to favour catalytic performance through high exposure of the active sites during catalytic reactions. Meanwhile, the Co_3_O_4_/ZrO_2_ catalyst (Figure 3h,i) formed a more compact ZrO_2_ surface with dispersed, perfectly shaped Co_3_O_4_ octahedra and a few distinguishable pores. This densely packed closed structure may limit the surface exposure and diffusion pathways, likely to contribute to a lower observed catalytic activity compared to the CeO_2_ catalytic system.

Energy‐dispersive X‐ray spectroscopy (EDS) map analysis was carried out to assess the elemental distribution within the composite catalysts. For the Co_3_O_4_/CeO_2_ system, the EDS map data reveal a homogeneous distribution of Co and Ce throughout the matrix, indicating uniform dispersion of Co_3_O_4_ across the CeO_2_ (Figure 3c–f). This suggests a well‐integrated heterostructure with strong interfacial contact between the two phases of the composite. Thus, this combination may be beneficial for the enhanced redox interactions and electronic transitions during catalysis. In the Co_3_O_4_/ZrO_2_ composite, the EDS analysis shows a different distribution pattern (Figure 3j–m). Well‐defined Co_3_O_4_ nano‐octahedra were primarily observed anchored on the ZrO_2_ substrate. In addition, a partial incorporation of cobalt into the ZrO_2_ matrix was also evident as trace levels of Co were detected within the ZrO_2_‐rich regions. These trace levels of cobalt inclusion can be attributed to the limited cation diffusion or interfacial doping, possibly due to ion migration, structural interaction during synthesis or thermal treatment. Such incorporation may induce lattice strain or alter the surface chemistry of ZrO_2_, although the overall cobalt distribution remained less uniform overall, compared to the CeO_2_‐based system.

**FIGURE 4:**
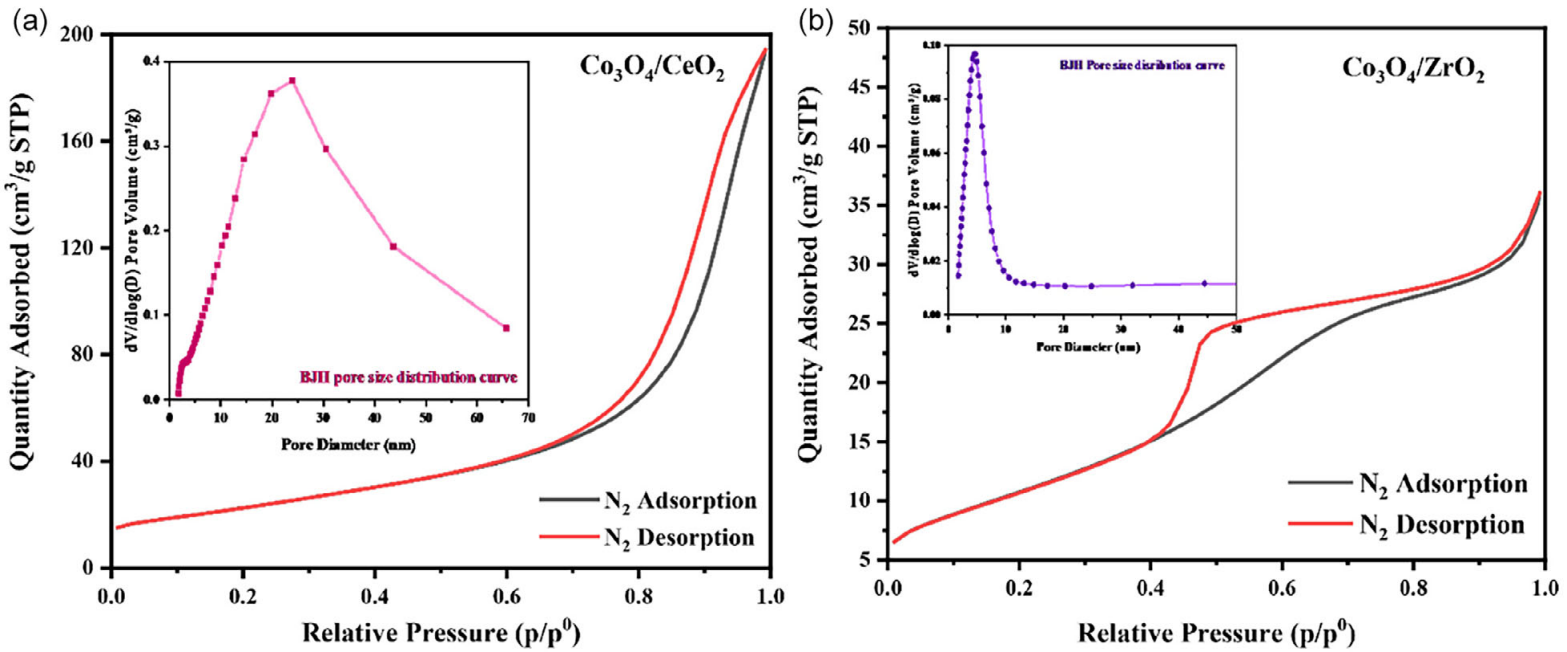
(a,b) Adsorption–desorption isotherms and BJH pore size distribution curves of Co_3_O_4_/CeO_2_ and Co_3_O_4_/ZrO_2_.

Nitrogen sorption measurements were used to study the surface area and porosity of the synthesised catalyst supports. Before the gas sorption analysis, samples were degassed at 200°C for 12 h to remove moisture and possible contaminants. Figure 4 gives the N_2_ adsorption–desorption isotherms and the corresponding Barrett–Joyner‐Halenda (BJH) pore size distribution curves of the Co_3_O_4_/CeO_2_ and Co_3_O_4_/ZrO_2_ catalysts. The Co_3_O_4_/CeO_2_ synthesised from the coprecipitation method shows Type IV isotherm behaviour, with H3 hysteresis loop, confirming the highly ordered mesoporous structure of the material [46, 47]. This is consistent with the presence of capillary condensation and slit like pores [48, 49]. The hysteresis loop of the Co_3_O_4_/CeO_2_ sample does not exhibit any regulating adsorption in the higher relative pressure range, indicating the presence of aggregated particles and nonuniform packing voids [47]. The rapid rise of the adsorbed quantity occurs at a relative pressure (P/P^0^) of 0.7 due to the filling of macropores formed by the aggregation of the catalyst particles [47, 50]. The BJH pore size distribution curve of Co_3_O_4_/CeO_2_ illustrates that the pore diameters are widely distributed in the range of 2–65 nm, with a maximum in the distribution curve at 24 nm.

Modified Co_3_O_4_/ZrO_2_ catalysts generated a Type V isotherm with H4 hysteresis loop, which reveals the composite nature of the catalyst support. The hysteresis loop indicates that mono and multilayer physisorption and capillary condensation take place after the initial stage of reversible micropore filling [46]. The shape of the highest point of the isotherm reveals that pore filling is ongoing, which may be due to the presence of macropores in the material or the packing voids of the nanoparticles. Furthermore, the H4‐type hysteresis loop confirmed the presence of aggregated crystals on the mesoporous supports, which is also evident in the SEM images of the Co_3_O_4_/ZrO_2_ samples [45]. BJH analysis of the Co_3_O_4_/ZrO_2_ data indicated that the pore sizes are scattered in the range of 2–44 nm, with a maximum in the distribution curve at 4.5 nm.

The Brunauer–Emmet–Teller (BET) surface area, total pore volume and average pore sizes of the prepared samples in the present study and relevant literature‐reported samples are given in Table 2. The Co_3_O_4_/CeO_2_ catalysts synthesised in this study show a comparatively high BET surface area relative to those reported in the literature. This improvement can be attributed to specific synthesis conditions and the actual molar ratio of individual components, which significantly influence surface area by affecting the nucleation, growth of the crystals and porosity enhancement during the catalyst material formation. The comparatively low BET surface area of the Co_3_O_4_/ZrO_2_ composite relative to Co_3_O_4_/CeO_2_ may account for its reduced catalytic activity observed in vanillyl alcohol oxidation in the present study.

**TABLE 2:** BET surface area, pore volume, and average pore diameter of different catalysts.

Sample	BET surface area, m^2^g^−1^	Total pore volume, cm^3^g^−1^	Average pore diameter, nm	Reference
Co_3_O_4_/CeO_2_	83	0.024	13.4	Present Study
Co_3_O_4_/ZrO_2_	40	0.010	5.1	Present Study
Co_3_O_4_/t‐ZrO_2_	24	0.022	3.7	[45]
Co_3_O_4_/m‐ZrO_2_	81	0.30	15.8	[45]
Co_3_O_4_/CeO_2_ (CeCo20‐CP)	79.4	—	—	[51]
Co_3_O_4_/CeO_2_ (CeCo20‐IM)	43.3	—	—	[51]
Co_3_O_4_/CeO_2_ (Co30Ce)	53	—	—	[52]
Co_3_O_4_/CeO_2_ (1:1)	41.54	0.41	—	[18]

**FIGURE 5:**
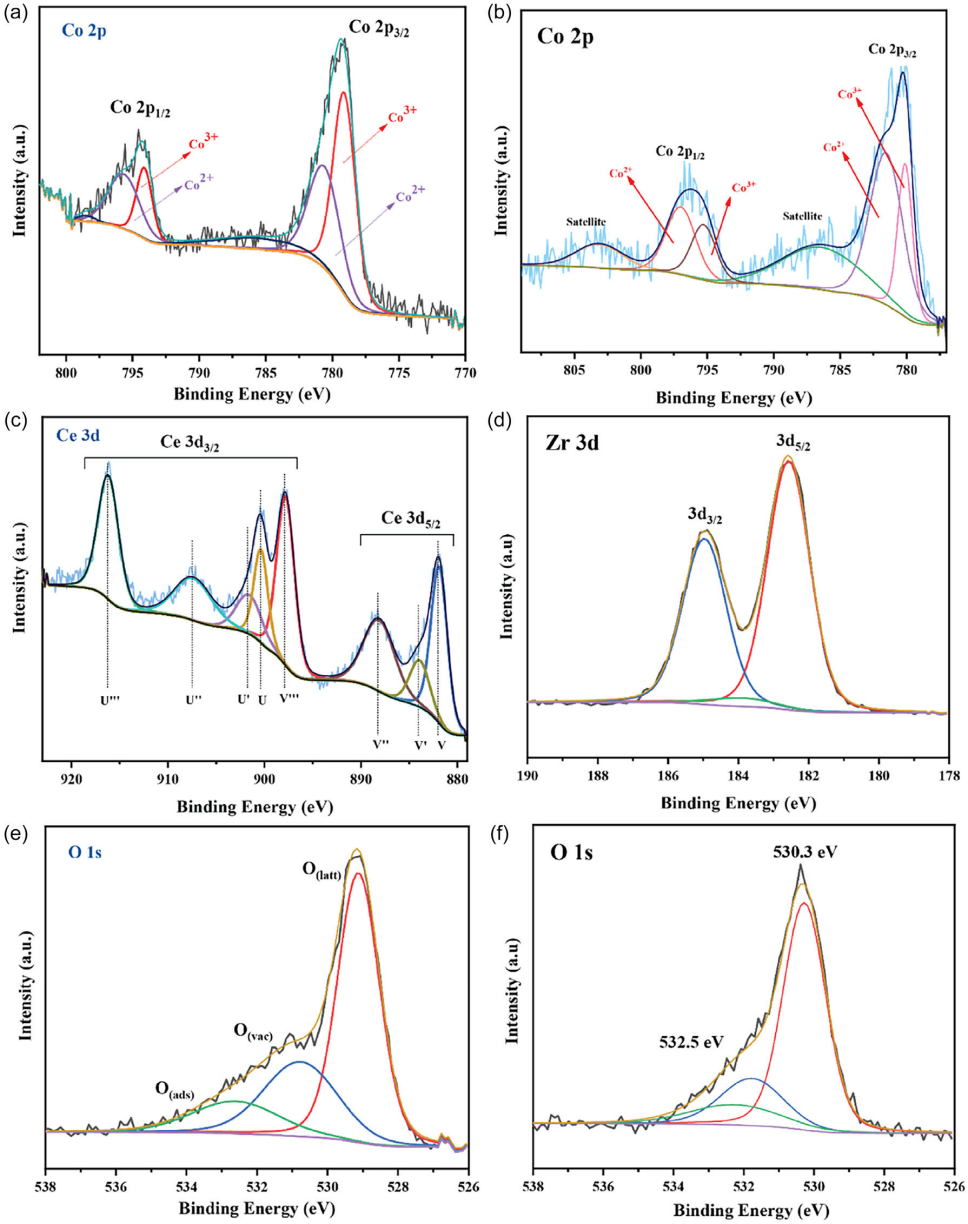
High‐resolution XPS spectra of (a) Co 2p in Co_3_O_4_/CeO_2_, (b) Co 2p in Co_3_O_4_/ZrO_2_, (c) Ce 3d in Co_3_O_4_/CeO_2_, (d) Zr 3d in Co_3_O_4_/ZrO_2_, (e) O 1s in Co_3_O_4_/CeO_2_, and (f) O 1s in Co_3_O_4_/ZrO_2_.

XPS survey scans confirmed the presence of Ce, Co, C, O and Zr, Co, O, C as the major elements of the Co_3_O_4_/CeO_2_ and Co_3_O_4_/ZrO_2_ catalysts, respectively (Figure S2). The Co 2*p* XPS spectrum of Co_3_O_4_/CeO_2_ (Figure 5a) features two main peaks in the 780 and 795 eV regions, corresponding to Co 2*p*_3/2_ and 2*p*_1/2_, respectively, along with two characteristic satellite peaks [48]. The lower energy region can be deconvoluted into two peaks at 779.1 and 780.7 eV, representing Co^3+^ and Co^2+^, respectively. Similarly, the high‐energy band could be deconvoluted into two peaks at 794.2 and 795.9 eV, indicating the presence of Co^3+^ and Co^2+^, respectively. In Figure 5b, the Co 2*p* spectrum of the Co_3_O_4_/ZrO_2_ catalyst exhibits two large peaks at binding energies of 780 and 796 eV, assigned to the Co 2*p*_3/2_ and 2*p*_1/2_ respectively [14]. Two satellite peaks were also identified at the positions of 786 and 803 eV, which are characteristic of the spinel Co_3_O_4_ [48]. These Co 2*p* peaks could be fitted and were assigned to surface Co^3+^ species (780.14, 795.35 eV) and Co^2+^ species (781.83, 797.05 eV).

As shown in Figure 5c, the Ce 3*d* core‐level XPS spectrum of Co_3_O_4_/CeO_2_ was deconvoluted into eight components, where the peaks labelled U′′′, V′′′, U′′, V′′, U, and V are assigned to the Ce^4+^ 3*d*_3/2_, Ce^4+^ 3*d*_5/2_, Ce^4+^ satellite, Ce^4+^ satellite, Ce^4+^ satellite, and Ce^4+^ satellite, respectively. Furthermore, the U′ and V′ represent the peaks of Ce^3+^ 3*d*_3/2_ and Ce^3+^ 3*d*_5/2_, confirming the presence of both Ce^3+^ and Ce^4+^ in the CeO_2_ matrix [26, 48]. The coexistence of both Ce^3+^ and Ce^4+^ oxidation states is indicative of charge imbalance within the lattice, which is often associated with the presence of oxygen vacancies [48]. The Zr 3*d* core spectrum of Co_3_O_4_/ZrO_2_ consisted of two distinct peaks, one at 182.5 eV and another at 184.8 eV, corresponding to 3*d*_5/2_ and 3*d*_3/2_ spin states of Zr, respectively (Figure 5d). The peak of binding energy 182.5 eV aligns with the Zr^4+^ oxidation state [53].

The O 1*s* core level spectrum gives three prominent oxygen peaks that correspond to three different types of oxygen atom coordination on the surface of the catalysts. The peak centred at 529.12 eV in Co_3_O_4_/CeO_2_ is attributed to surface lattice oxygen bound to the metals or lattice (Figure 5e). The second peak, located at 530.8 eV, corresponds to low‐coordinated oxygen species adsorbed on surface oxygen vacancies. The third peak, appearing around 532.7 eV, is associated with chemisorbed oxygen species on the catalyst [26, 48]. In the Co_3_O_4_/ZrO_2_ system, the peak centred at 530.3 eV is characteristic of surface lattice oxygen atoms, and the peak centred at 531.8 eV can be assigned to oxygen species adsorbed over the surface oxygen vacancies (Figure 5f). The third peak at 532.3 eV is due to the chemisorbed oxygen atoms on the surface of the catalyst [54]. Quantitative XPS analysis confirmed that the proportion of surface oxygen vacancies in Co_3_O_4_/CeO_2_ (28%) was considerably greater than that in Co_3_O_4_/ZrO_2_ (16%), indicative of a higher density of defect sites on the Co_3_O_4_/CeO_2_ surface.

**FIGURE 6:**
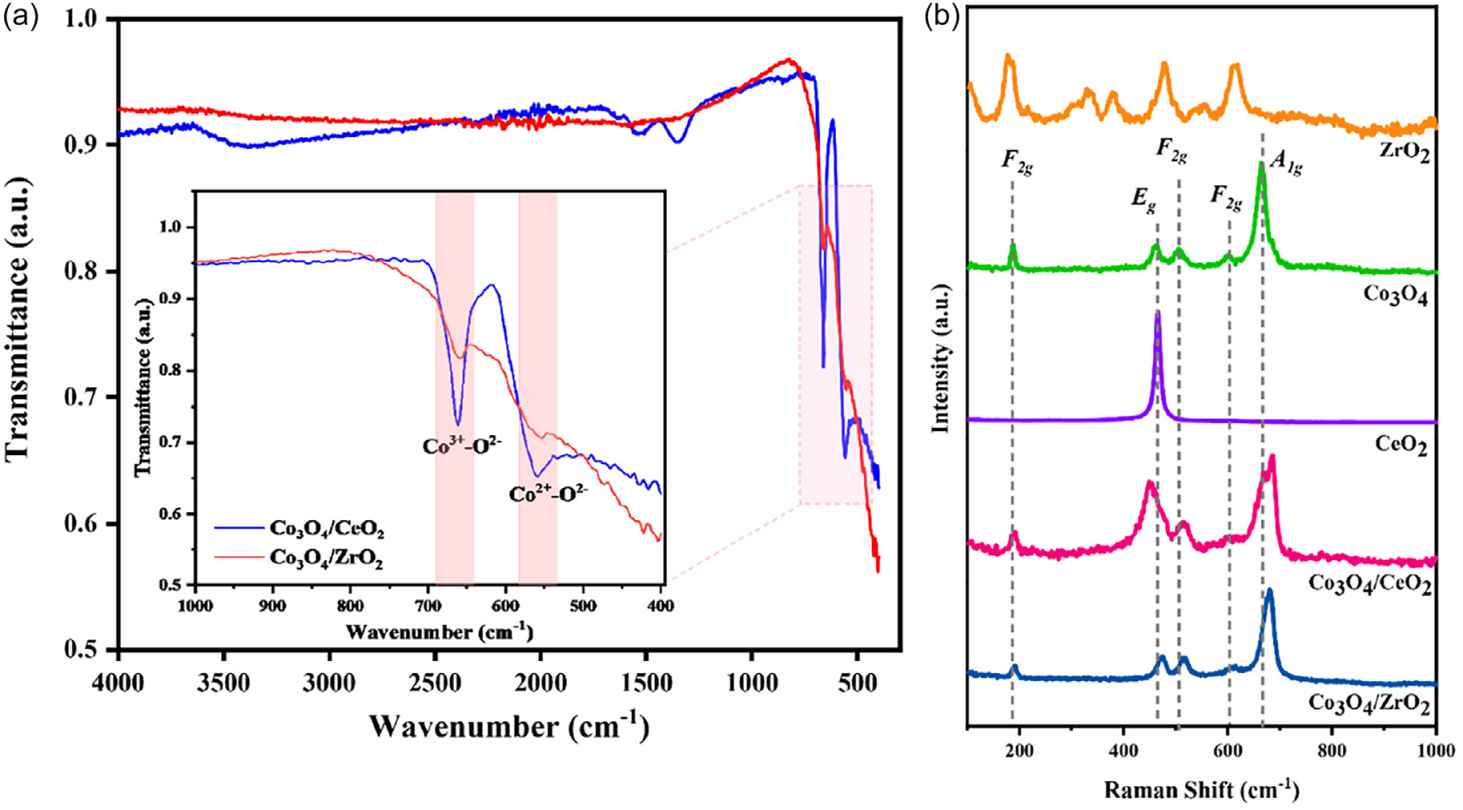
(a) The ATR‐FTIR spectra of Co_3_O_4_/CeO_2_ and Co_3_O_4_/ZrO_2_ composites. (b) The Raman spectra of bare ZrO_2_, bare CeO_2_, bare Co_3_O_4_, Co_3_O_4_/CeO_2_ composite, and Co_3_O_4_/ZrO_2_ composite.

As displayed in Figure 6a, the FTIR spectra of Co_3_O_4_/CeO_2_ and Co_3_O_4_/ZrO_2_ composites contained characteristic Co–O vibrational bands in the 560–670 cm^−1^ range, with a strong peak around 561 cm^−1^, which is associated with Co^2+^O^2−^ and another around 663 cm^−1^, where the Co^3+^‐O^2‐^ peak can be identified [18]. High‐intensity bands in Co_3_O_4_/CeO_2_ suggest a stronger metal–support interaction of the composite. Additionally, the intense bands at 3400 cm^−1^ in Co_3_O_4_/CeO_2_ indicate the −OH and water adsorption bands, suggesting a higher surface hydroxyl density and oxygen vacancy concentration, consistent with the redox‐active nature of ceria [18]. In contrast, Co_3_O_4_/ZrO_2_ shows relatively less pronounced defect‐related features, reflecting the lower reducibility of ZrO_2_.

To obtain complementary structural information, Raman spectroscopy was performed on both catalysts (Figure 6b). The Raman spectrum of pristine Co_3_O_4_ consisted of five distinct peaks, characteristic of the spinel crystal structure. These peaks correspond to the A_1g_, 3 × F_2g_ and E_g_ vibrational modes, typically observed around 665, 600, 508, 465 and 192 cm^−1^ respectively [45, 55]. Bare CeO_2_ displayed a prominent Raman band at around 465, attributed to the F_2g_ mode of the cubic fluorite structure [51]. In the Co_3_O_4_/CeO_2_ composite structure, all five vibrational modes of the Co_3_O_4_ spinel structure were retained. However, the peaks were significantly broadened and slightly shifted towards higher wavenumbers, indicating strong interaction between the two phases or lattice strain due to the incorporation of smaller cations, as evidenced by the XRD analysis [56]. The F_2g_ mode of CeO_2_ at 465 cm^−1^ was not distinguishable, probably due to overlap and masking by the surface‐dominant Co_3_O_4_ bands. Similarly, the Raman spectrum of the Co_3_O_4_/ZrO_2_ catalyst exhibits all the vibrational bands of Co_3_O_4_, with slight shifts towards higher wavenumbers. The typical six vibrational bands of tetragonal ZrO_2_ (A_1g_ + 2B_1g_ + 3E_g_) may be masked by the Co_3_O_4_ bands in the composite [57]. The peaks in the Co_3_O_4_/ZrO_2_ catalyst were much narrower than those in the Co_3_O_4_/CeO_2_ system, possibly due to the less pronounced interaction between Co_3_O_4_ and ZrO_2_ in the composite system [45].

### 3.2 Activity Test of Co_3_O_4_/CeO_2_ and Co_3_O_4_/ZrO_2_ Catalysts for Oxidation of Vanillyl Alcohol

**SCHEME 1:**
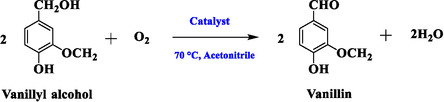
Oxidation of vanillyl alcohol.

The catalytic activities of Co_3_O_4_/CeO_2_ and Co_3_O_4_/ZrO_2_ systems for the valorisation of lignin derivatives were investigated using vanillyl alcohol as a model compound. As displayed in Scheme 1, the hydroxyl group of the vanillyl alcohol can be selectively oxidised to the aldehyde under mild reaction conditions using modified heterogeneous catalysts.

**FIGURE 7:**
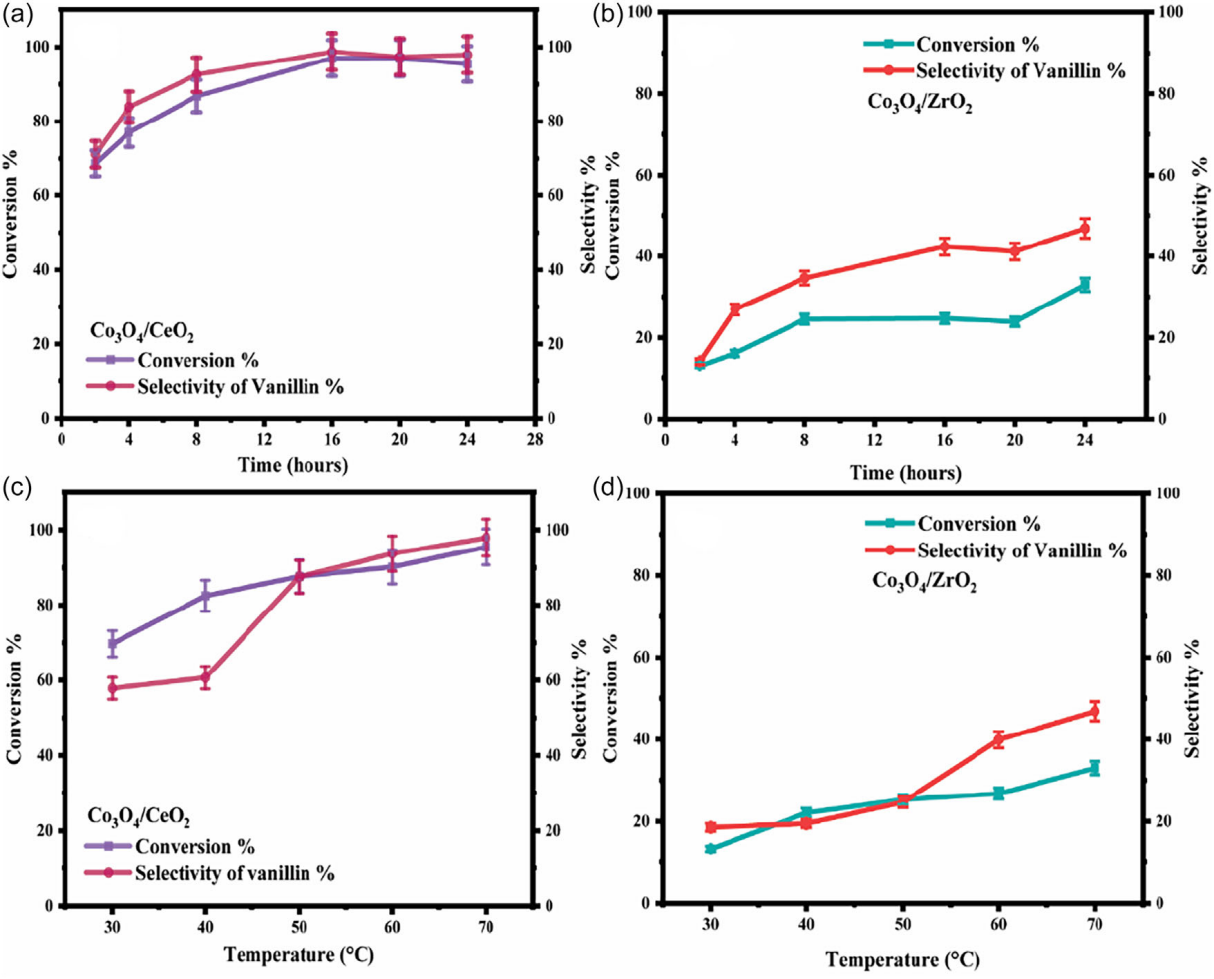
The effect on the reaction time with (a) Co_3_O_4_/CeO_2_ and (b) Co_3_O_4_/ZrO_2._ The effect of reaction temperature with (c) Co_3_O_4_/CeO_2_ and (d) Co_3_O_4_/ZrO_2_.

Acetonitrile was chosen as the solvent medium for the reaction due to its high polarity and reasonably high boiling point (82°C) [6, 12, 58]. Reaction time and temperature were optimised for the oxidation reaction for both catalysts. Generally, the Co_3_O_4_/CeO_2_ catalyst exhibited superior activity relative to the Co_3_O_4_/ZrO_2_ system at all tested reaction temperatures and times. The effect of time on reaction conversion and product selectivity was explored from 2 to 24 h for both catalysts (Figure 7a,b). The conversion efficiency of the substrate with Co_3_O_4_/CeO_2_ catalyst increased significantly from 68% at 2 h to 97% at 16 h, after which it remained constant up to 24 h. Similarly, the selectivity improved from 71% to 98% over the same period, with no further significant enhancement beyond 16 h. Consequently, 16 h was determined to be the optimal reaction time for the Co_3_O_4_/CeO_2_ catalyst, achieving both high conversion and selectivity. In contrast, the Co_3_O_4_/ZrO_2_ catalyst exhibited considerably lower activity. The conversion gradually increased from 13% at 2 h to a maximum of only 32% at 24 h. The selectivity also remained low throughout the reaction, reaching a maximum of just 46%. The initial low selectivity can be attributed to competing parallel and consecutive oxidation pathways at high reactant concentrations. As the reaction proceeds, changes in the catalyst surface coverage and adsorption behaviour moderate the reactivity of active sites, suppressing over‐oxidation and improving selectivity. It is well established that adsorption strength and surface site availability play a critical role in determining product distribution in heterogeneous catalysis, and partial blocking of highly active sites can enhance selectivity towards the desired product [59, 60].

The catalytic performances of the two systems were evaluated over a temperature range of 30°C–70°C (Figure 7c,d). The maximum temperature was limited to 70°C to avoid exceeding the solvent's boiling point during the reaction. The highest conversion efficiency and selectivity were observed at 70°C for both catalysts, and this was consequently chosen as the optimal reaction temperature in further experiments. Notably, under these conditions, the Co_3_O_4_/CeO_2_ system still achieved near quantitative conversion and selectivity.

**FIGURE 8:**
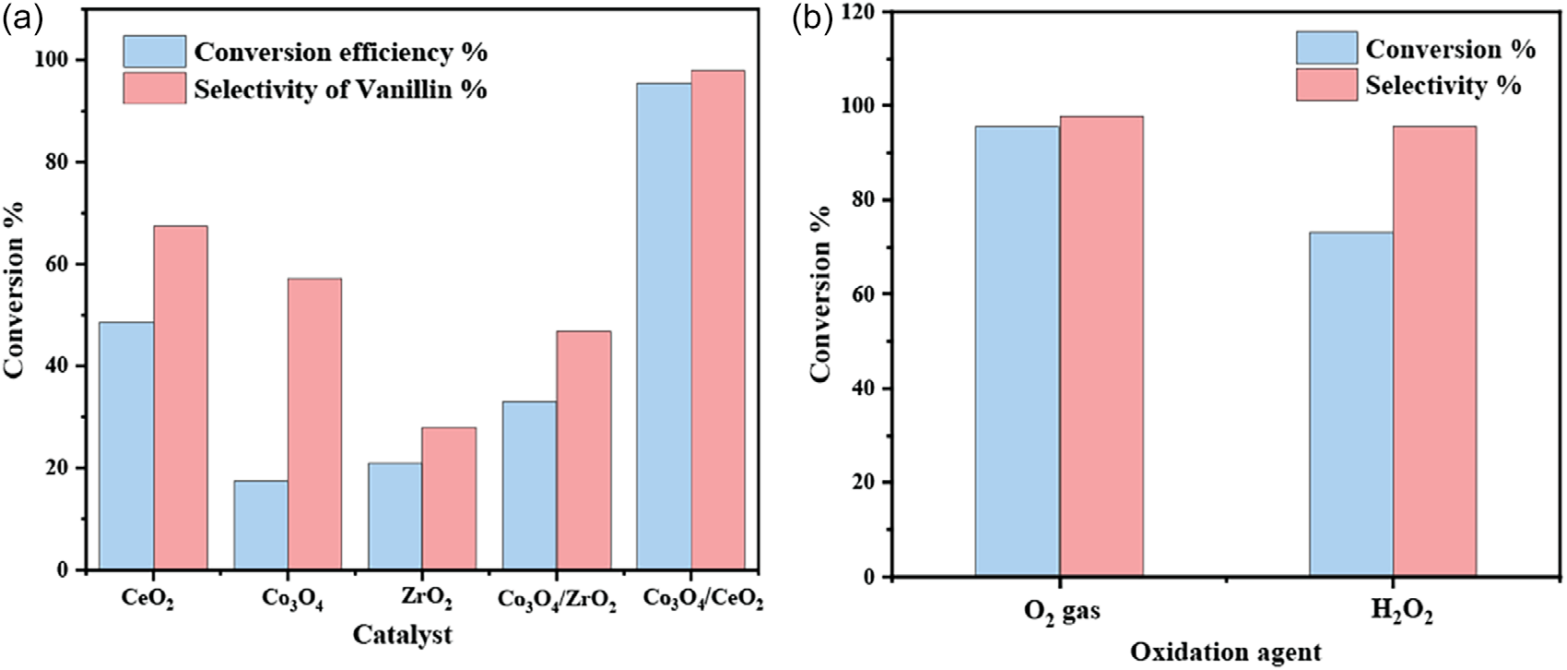
The effect of (a) catalyst support and (b) the oxidant on the vanillyl alcohol oxidation reaction at 70°C for 24 h.

The catalytic activity of the bare CeO_2_, ZrO_2_, and Co_3_O_4_ materials was also evaluated for the vanillyl alcohol oxidation reaction, as shown in Figure 8a. Pure CeO_2_ exhibited significantly higher conversion compared to the ZrO_2_ catalyst. Co_3_O_4_ alone imparted very low conversion efficiency for the reaction. However, the Co_3_O_4_/CeO_2_ composite catalyst demonstrated superior performance relative to bare CeO_2_, ZrO_2_, and Co_3_O_4_, achieving the highest conversion and selectivity. These results suggest, especially when taking the XPS results of Figure 5 into account, that the enhanced activity of the Co_3_O_4_/CeO_2_ can be attributed to the increased number of oxygen vacancies and active sites, which are more abundant in the CeO_2_ composite than in ZrO_2_. A CeO_2_ supported composite may influence the reversible redox cycling of cobalt ions, which can also be a key factor underlying the enhanced activity. Results of the reactant's adsorption–desorption study (Table S1) revealed that the Co_3_O_4_/CeO_2_ catalyst possesses almost a five‐fold greater affinity towards the vanillyl alcohol molecule than Co_3_O_4_/ZrO_2_, suggesting that this enhanced adsorption capability might also facilitate its superior performance in the oxidation of vanillyl alcohol. Furthermore, the presence of larger mesopores in Co_3_O_4_/CeO_2_ can be expected to facilitate more efficient diffusion of reactants to the active sites compared to the smaller mesopores in Co_3_O_4_/ZrO_2_.

The impact of the oxidizing agent was analysed (Figure 8b) using molecular oxygen and H_2_O_2_ over a Co_3_O_4_/CeO_2_ catalyst, and the results confirmed molecular oxygen as the preferred oxidation agent for vanillyl alcohol oxidation. This may be because of the higher reactivity and stronger oxidative potential of molecular oxygen compared to H_2_O_2_. Molecular oxygen can serve as a superior direct and indirect electron acceptor, with high mobility and reactivity, allowing it to readily participate in the oxidation pathway [61]. To further explore the influence of metal composition and thermal treatment on catalytic performance, Co_3_O_4_/CeO_2_ catalysts with varying metal ratios were synthesised. Additionally, the calcination temperature was also varied to evaluate the effect on the catalytic activity. The results of these studies are presented in Figure S3.

**FIGURE 9:**
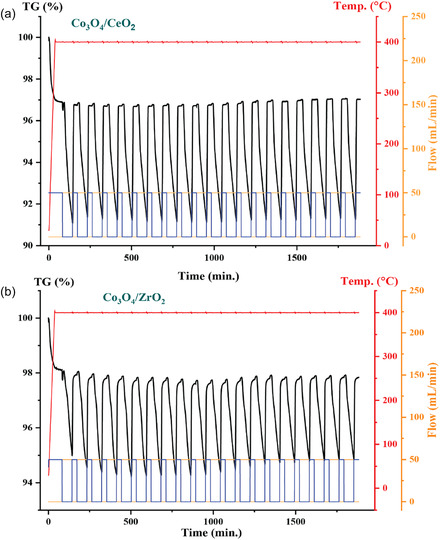
Cyclic thermal gravimetric analysis curves for (a) Co_3_O_4_/CeO_2_ and (b) Co_3_O_4_/ZrO_2_.

As is evident from the results of the oxidation reaction with both the Co_3_O_4_/CeO_2_ catalyst and Co_3_O_4_/ZrO_2_ catalyst, the CeO_2_‐Co_3_O_4_ heterojunction is very effective for vanillyl alcohol oxidation. Pure CeO_2_ is well known for its high OSC, which arises from facile transformation between CeO_2_ and CeO_2‐x_ [62]. Therefore, ceria is predominantly employed in catalysis as an efficient oxygen carrier [62]. To explain the catalytic performances of the two catalysts, quantitative OSC measurements were carried out using cyclic thermogravimetric analysis (Figure 9). This measured reversible oxygen uptake tracks directly with oxidation rates, establishing lattice oxygen availability and redox flexibility as the key descriptors of catalytic activity [63]. The OSC for the Co_3_O_4_/CeO_2_ is **≈**4375 μmol O/g, whereas the Co_3_O_4_/ZrO_2_ catalyst exhibits a significantly lower OSC of around 1875 μmol O/g. This notably higher OSC of the Co_3_O_4_/CeO_2_ system indicates an enhanced ability to store and release oxygen through the Ce^4+^/Ce^3+^ redox cycle, which facilitates the continuous replacement of reactive oxygen species (ROS) on the catalyst surface. This higher oxygen mobility promotes efficient oxidation of vanillyl alcohol over the Co_3_O_4_/CeO_2_ catalyst, while the limited OSC of Co_3_O_4_/ZrO_2_ restricts the supply of ROS, leading to the lower catalytic activity. On the basis of this evidence, Co_3_O_4_/CeO_2_ has been chosen as the best catalytic system for selective vanillyl alcohol oxidation and further catalytic experiments were carried out only for the Co_3_O_4_/CeO_2_ system.

**FIGURE 10:**
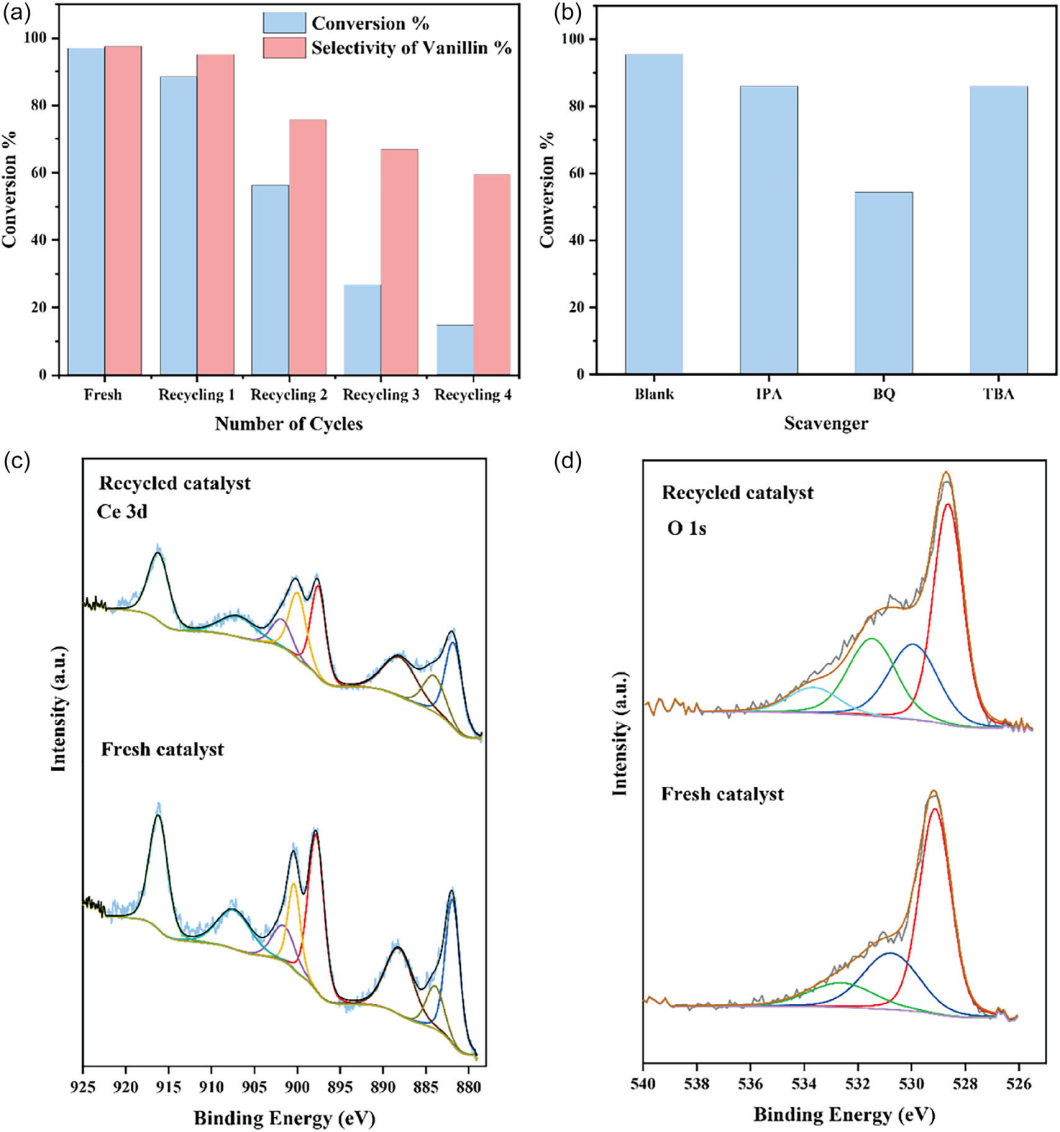
(a) Reusability of the Co_3_O_4_/CeO_2_ catalyst over 4 cycles of vanillyl alcohol oxidation at 70°C for 16 h with 20 mg of catalyst. (b) Quenching studies with IPA, BQ, and TBA for vanillyl alcohol oxidation with Co_3_O_4_/CeO_2_ catalyst at 70°C for 16 h. (c) Core level XPS spectra of Ce 3d for recycled and fresh Co_3_O_4_/CeO_2_ catalyst. (d) Core level XPS spectra of O 1s of recycled and fresh Co_3_O_4_/CeO_2_ catalyst.

The reusability of the catalyst is an essential factor in the practical application of heterogeneous catalysis. Hence, the recyclability of the Co_3_O_4_/CeO_2_ catalyst was evaluated towards the oxidation of vanillyl alcohol to understand the stability of the catalyst. After the first cycle, the catalyst was recovered by centrifugation at 3800 rpm for 5 min, washed sequentially with DI water and ethanol, and then dried at 60°C overnight. Dried catalysts were used for the subsequent recycling without further activation. Due to the small amounts of catalysts used in each run, recovery limited the study to 4 reuse cycles. Within this limited dataset, a clear and consistent trend in catalytic performance was observed. As illustrated in Figure 10a, the conversion efficiency of the reaction decreased from fresh samples to the fourth reuse cycle. The selectivity for vanillin also reduced from 99% to 60% over the cycles.

To understand the observed deactivation, XPS analysis was performed on the recycled catalysts after the 4^th^ cycle to observe the changes in the surface after the reaction. The Ce 3*d*, Co 2*p*, O 1*s*, and C 1*s* core level spectra of the reused catalysts exhibited slight shifts towards lower binding energies compared with the fresh catalysts (Figure S4). This suggests changes in the composition of surface elements resulting from alterations in the electron density around the surface metal groups due to the reaction. The partial reduction of Ce^4+^ to Ce^3+^ and Co^3+^ to Co^2+^ and the generation of oxygen vacancies during the reaction may be responsible for the changes in the electron density of the surface metals and oxygen sites [64]. Post reaction XPS analysis shows no substantial loss of Co species, suggesting that extensive metal leaching is unlikely to be the dominant deactivation pathway under the applied reaction conditions.

A new peak appeared in the high‐resolution O 1*s* XPS spectra (Figure 10d) at around 533 eV in the recycled catalyst, which could be attributed to the presence of hydroxyl groups adsorbed to the catalyst surface. This supports the formation of water during the reaction, blocking (poisoning) the active sites of the catalyst by sticking onto the surface [65]. Under wet conditions, active sites may readily aggregate while decreasing the specific surface area [66]. Furthermore, the signal for the C 1*s* has increased in the reused catalyst (Figure S4), which indicates the presence of carbonaceous byproducts (coke) deposited on the catalyst surface, blocking surface sites, clogging the pores and reducing the selectivity for vanillin. HR‐TEM images of the spent catalyst (Figure S5) confirmed these changes, showing the presence of surface adsorbed species that were not observed in the fresh sample. These species are likely reaction intermediates or byproducts that can poison the active sites and suppress the activity [66].

Furthermore, to explain the decreased catalytic activity observed during the 4^th^ cycle, a morphological characterisation for the recycled catalysts was conducted (Figures S6). The original well‐defined porous structure of the catalyst deteriorated after repeated use, and the particles appeared to be aggregated. These changes are indicative of surface reconstruction and particle sintering under repeated redox conditions [66]. This reduces the active sites and effective surface area of the catalyst, while contributing to the catalyst deactivation. Overall, the catalyst deactivation can be attributed to a combination of surface poisoning by hydroxyl groups and carbonaceous deposits, together with structural changes such as aggregation and partial reduction of active species. From a practical standpoint, catalyst regeneration can be achieved by mild calcination in air to remove surface carbon species and hydroxyl groups to restore active sites, as commonly employed for Co‐based mixed oxide catalysts. However, repeated thermal treatments may also promote sintering, which could limit the long‐term stability of the catalyst. Alternatively, oxidative washing or low‐temperature calcination could partially restore the activity by reopening oxygen vacancy sites [67, 68].

Reaction quenching tests were conducted using different scavenging agents to investigate the mechanism of reaction and the role of ROS involved in vanillyl alcohol oxidation with a Co_3_O_4_/CeO_2_ catalyst. Isopropanol (IPA) and tert‐butyl alcohol (TBA) were utilised to quench HO^•^ radicals, while benzoquinone (BQ) was used to quench superoxide radicals (O_2_^•−^) [7, 18]. The conversion efficiency of the reaction was reduced to 54% with the addition of a controlled amount of BQ, whereas it remains in the range of 86% in the presence of IPA and TBA (Figure 10b). From the results of Figure 10b, it may be concluded that hydroxyl radicals and the formation of hydroxyl radicals from the catalyst are a very minor factor in the overall reaction mechanism. These results indicate that instead, superoxide radicals have a considerable effect on the oxidation reaction with Co_3_O_4_/CeO_2_ catalyst. EPR experiments were also carried out to highlight the origin of this radical formation in this material.

**FIGURE 11:**
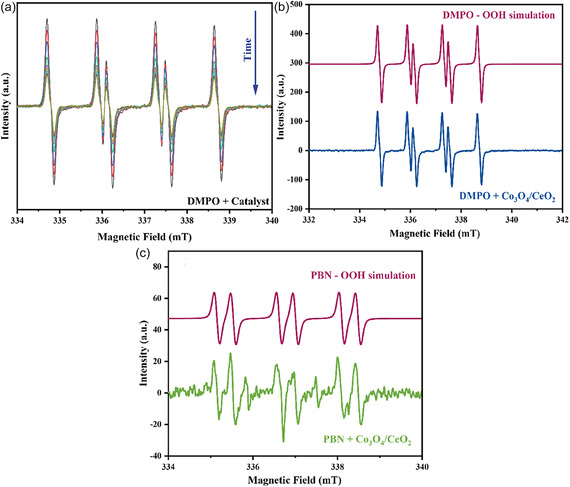
(a) Time resolved X‐band EPR spectra of DMPO spin adducts generated from Co_3_O_4_/CeO_2_ catalysts in DMSO at 70°C. (b) Experimental and simulated EPR spectra of the DMPO‐OOH adduct; *g* = 2.0065, A_*N*_ = 1.385 mT, A_H1_ = 0.1170 mT, A_H2_ = 0.0845 mT. (c) Experimental and simulated EPR spectra of the PBN‐OOH adduct generated from Co_3_O_4_/CeO_2_ catalyst in acetonitrile at 70°C; *g* = 2.0066, A_*N*_ = 1.475 mT, A_H_ = 0.386 mT.

EPR spin trapping studies of the Co_3_O_4_/CeO_2_ system that used the DMPO spin trap in DMSO revealed the presence of the superoxide adduct only (Figure 11a,b). The intensity of the adduct spectrum decreased with time, which is consistent with the limited lifetime of the adduct and consumption of the DMPO. There was no evidence of HO^•^ or ^•^CH_3_ adducts (from the reaction of HO^•^ with DMSO) of DMPO, consistent with the bulk reaction quenching results of Figure 10b.

When PBN was used as the spin‐trapping agent in EPR, the signal was dominated by the PBN‐OOH adduct (Figure 11c). While the residual weak 3‐line signal did not match any expected adducts, this is likely due to decomposition products of the PBN. The spin trapping experiments, in combination with the reaction quenching studies, strongly support a dominant role of superoxide in the vanillyl alcohol oxidation over the Co_3_O_4_/CeO_2_ catalyst. Hydroxyl radicals were not detected (directly or indirectly) and consequently are not implicated in the reaction mechanism.

**FIGURE 12:**
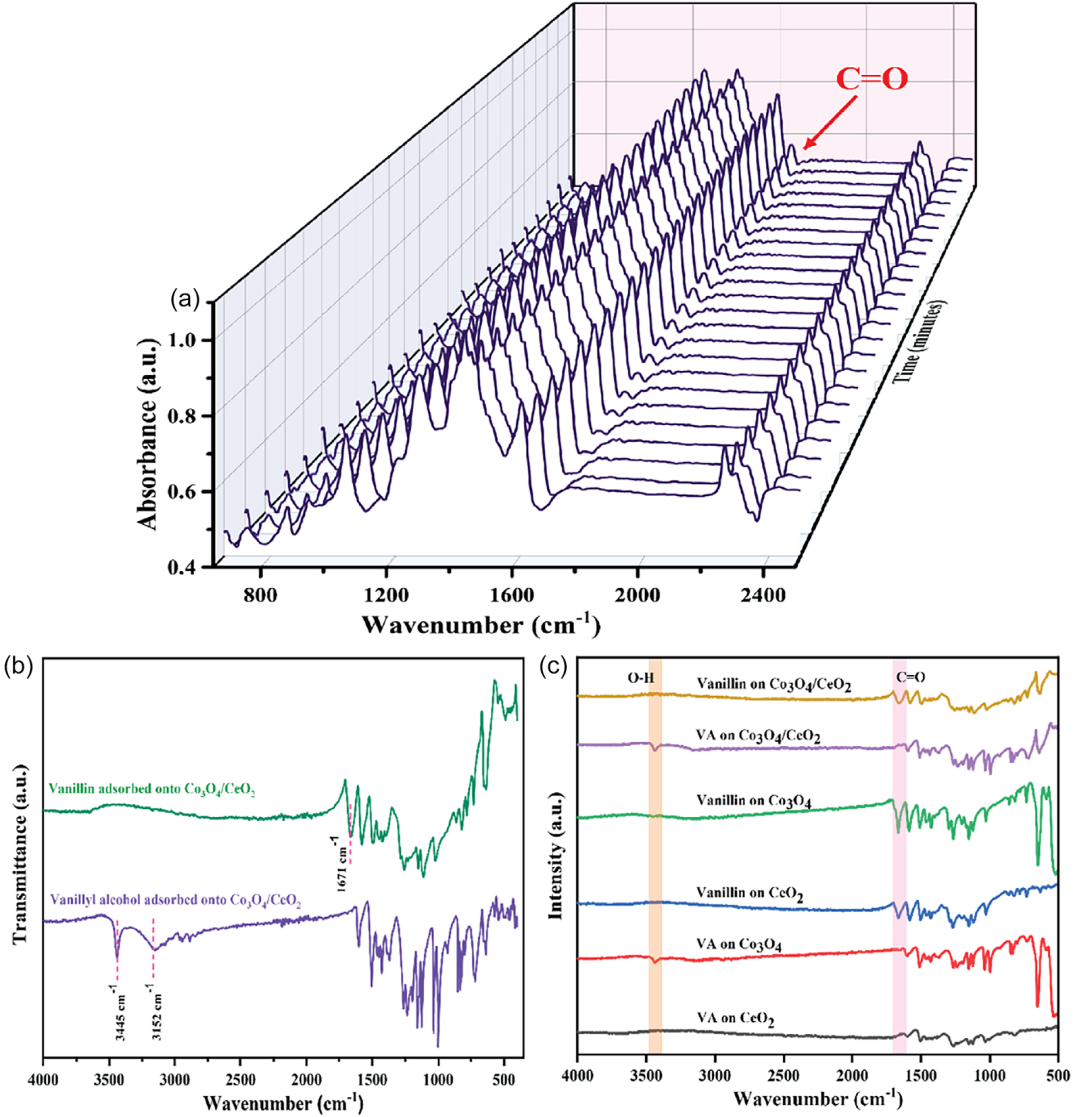
(a) In situ DRIFTS analysis of vanillyl alcohol oxidation with Co_3_O_4_/CeO_2_ catalyst at 70°C under atmospheric oxygen in a commercial in situ DRIFTS cell. (b) The ATR‐FTIR spectra of vanillyl alcohol and vanillin adsorbed onto the Co_3_O_4_/CeO_2_ catalyst. (c) The ATR‐FTIR spectra of standard vanillyl alcohol and vanillin over bare CeO_2_, bare Co_3_O_4_ and Co_3_O_4_/CeO_2_ composite.

To examine the selective formation of vanillin over the Co_3_O_4_/CeO_2_ catalyst, time‐resolved in situ DRIFTS studies were conducted. Standard samples of vanillyl alcohol and vanillin were first adsorbed onto the Co_3_O_4_/CeO_2_ catalyst surface to identify the characteristic FTIR bands of the reactant and product before the DRIFTS study. In Figure 12b, the characteristic O–H stretching vibrations in vanillyl alcohol were observed at 3445 and 3152 cm^−1^; while the C=O stretching vibration of vanillin was visible at 1671 cm^−1^. These signatures in the spectra enable clear differentiation between the reactant and product.

Displayed in Figure 12a, the gradual formation of a distinct carbonyl band (C=O) at 1671 cm^−1^ over time signifies the selective formation of vanillin. The O–H stretch at 3450 cm^−1^ persisted over time as the reaction proceeded, indicating formation of water occurred as a side product. Other than the growth of the C=O band, no other significant peak changes were observed, confirming that no additional byproducts formed or were adsorbed onto the catalyst surface. Initially, the adsorption of vanillyl alcohol molecules preferentially occurs on the Co_3_O_4_ sites in the composite. A distinct O–H stretching band of vanillyl alcohol at ∼3400 cm^−1^ is observed only on Co_3_O_4_, whereas no corresponding feature is detected on CeO_2_ (Figure 12c). This suggests a stronger interaction of the reactant with Co_3_O_4_ sites in the composites. In the product spectra, the C=O stretching band at 1671 cm^−1^ exhibits significantly higher intensity on Co_3_O_4_ compared to CeO_2_, further supporting that Co_3_O_4_ provides more favourable sites for the adsorption and subsequent reaction, while CeO_2_ supports the reaction mechanism by making more active oxygen species for the reaction, as confirmed by the OSC data of Figure 9a.

**FIGURE 13:**
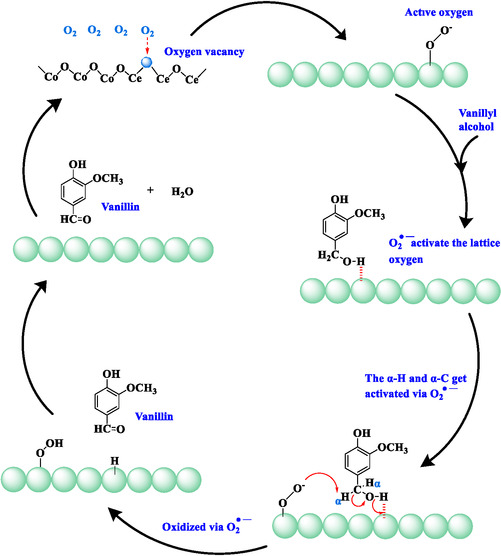
Suggested mechanism for vanillyl alcohol oxidation via Co_3_O_4_/CeO_2_ catalyst under thermal conditions.

Based on the results of the EPR analysis and radical‐quenching experiments, superoxide radicals were identified as the primary reactive species responsible for the oxidation of vanillyl alcohol. Therefore, a radical‐mediated mechanism for vanillyl alcohol oxidation catalysed by a Co_3_O_4_/CeO_2_ catalyst can be proposed, as shown in Figure 13. Similar radical‐mediated mechanisms for oxidation reactions are widely reported in literature studies [69, 70]. Initiated by adsorption of molecular O_2_ onto oxygen vacancies in the Co_3_O_4_/CeO_2_ system, the chemisorbed molecular O_2_ is activated to yield ROS (O_2_^•−^) by abstracting an electron from the catalyst system (redox cycle of Ce^3+^ to Ce^4+^ and Co^2+^ to Co^3+^) [18, 65, 71].

These ROS activate the lattice oxygen, enabling the adjacent surface basic oxygen sites to coordinate with the proton from the alcoholic hydroxyl group of vanillyl alcohol by weakening the O–H bond [65]. With that occurring, the α‐C and α‐H are activated and oxidised via the surface‐bound superoxides, while dissociating the O–H bond. Surface‐generated OH species may yield water as a byproduct at the end of the mechanism. This could be confirmed via the new O 1s peak that appeared in the XPS spectrum of the reused catalysts. With the desorption of generated vanillin molecules from the catalyst surface, lattice oxygen of the catalyst may be replenished from the active oxygen species.

## 4 Conclusion

In this study, Co_3_O_4_/CeO_2_ and Co_3_O_4_/ZrO_2_ nanocomposites were synthesised by the coprecipitation method as heterogeneous catalysts for the oxidation of vanillyl alcohol with O_2_ at atmospheric pressure. In‐depth characterizations correlated the catalytic activity with the physicochemical and structural features of the two composites. XRD, SAED, and XPS analyses confirmed the successful formation of Co_3_O_4_/CeO_2_ and Co_3_O_4_/ZrO_2_ systems, while the SEM and TEM analyses revealed the morphological features of the two samples. Quantitative OSC measurements for Co_3_O_4_/CeO_2_ and Co_3_O_4_/ZrO_2_ composites were **≈**4375 and 1875 μmol O/g, respectively, and confirmed the greater lattice oxygen availability and redox flexibility of the Co_3_O_4_/CeO_2_ system. N_2_ sorption measurements revealed the higher surface area and porosity of the Co_3_O_4_/CeO_2_ system relative to the Co_3_O_4_/ZrO_2_ system. The results of the catalytic activity tests under atmospheric pressure, with varied temperatures and reaction times, established Co_3_O_4_/CeO_2_ as a superior catalyst for vanillyl alcohol oxidation. Mechanistic studies were conducted using quenching studies, DRIFTS and EPR experiments to define a detailed proposed reaction pathway for vanillyl alcohol oxidation with Co_3_O_4_/CeO_2_ catalyst. The EPR results revealed that surface superoxide (O_2_^•−^) species are the dominant ROS that govern the oxidation mechanism. DRIFTS studies verified vanillin as the sole organic product, with water formed as the only side product, demonstrating the high selectivity of product formation. FTIR studies further exhibited that vanillyl alcohol preferentially adsorbs on Co_3_O_4_ active sites within the composite structure. Meanwhile, CeO_2_ plays a crucial role by enhancing oxygen availability, as evidenced by the quantitative OSC measurements. The stability of the Co_3_O_4_/CeO_2_ system was checked through recycling tests, and a decrease in catalytic activity observed during the fourth cycle was explained by partial deterioration of the original porous structure, particle aggregation, coke formation, and catalyst wetting due to surface hydroxyl groups via XPS and morphological analysis. Overall, this study provides a systematic structure–activity comparison of two composite catalysts under mild reaction conditions and elucidates the mechanistic pathway governing the selective oxidation of vanillyl alcohol to vanillin.

## Supporting Information

Additional supporting information can be found online in the Supporting Information section.

## Funding

This work was supported by the Australian Research Council (DP210103357).

## Conflicts of Interest

The authors declare no conflicts of interest.

## Supporting information

Supplementary Material

## Data Availability

The data that supports the findings of this study are available in the supplementary material of this article.
