# Supplementary material for: Co3O4/CeO2 and Co3O4/ZrO2 Composites as Heterogeneous Catalysts for Selective Aerobic Oxidation of Vanillyl Alcohol
Source: ChemistryOpen. 2026 May 14;15(5):e70228. doi: 10.1002/open.70228 (PMC13176639; doi:10.1002/open.70228)
Supplement: Supplementary file 1 — Supplementary Material [file OPEN-15-e70228-s001.pdf]

Supporting Information

Co_3O_4/CeO_2 and Co_3O_4/ZrO_2 Composites as Heterogeneous Catalysts for Selective Aerobic Oxidation of Vanillyl Alcohol

Hashini T. Abeyrathna[1], Chamodi L. Fernando Thibiripalage[1], Huai Yong Zhu[1], Yichao Jin[1], Aaron Micallef[2], Eric R. Waclawik[1*]

[1] School of Chemistry and Physics
Queensland University of Technology
Brisbane, QLD 4001, Australia

[2] Central Analytical Research Facility (CARF)
Queensland University of Technology
Brisbane, QLD 4001, Australia

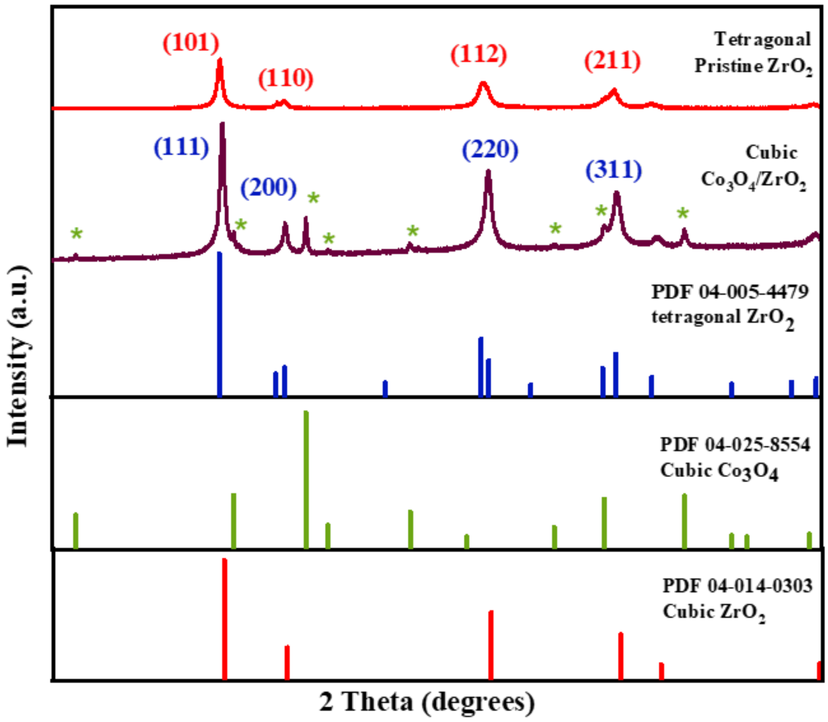

Figure S1: Powder XRD patterns of pristine ZrO_2 and Co_3O_4/ZrO_2. Vertical dropline diffraction patterns representing the reference PDF data of tetragonal ZrO_2, Cubic Co_3O_4 and Cubic ZrO_2. The diffraction pattern of pristine ZrO_2 matches with Tetragonal ZrO_2. In the composite sample, the peaks marked with stars (*) correspond to cubic Co_3O_4, while the remaining reflections are attributed to cubic ZrO_2.

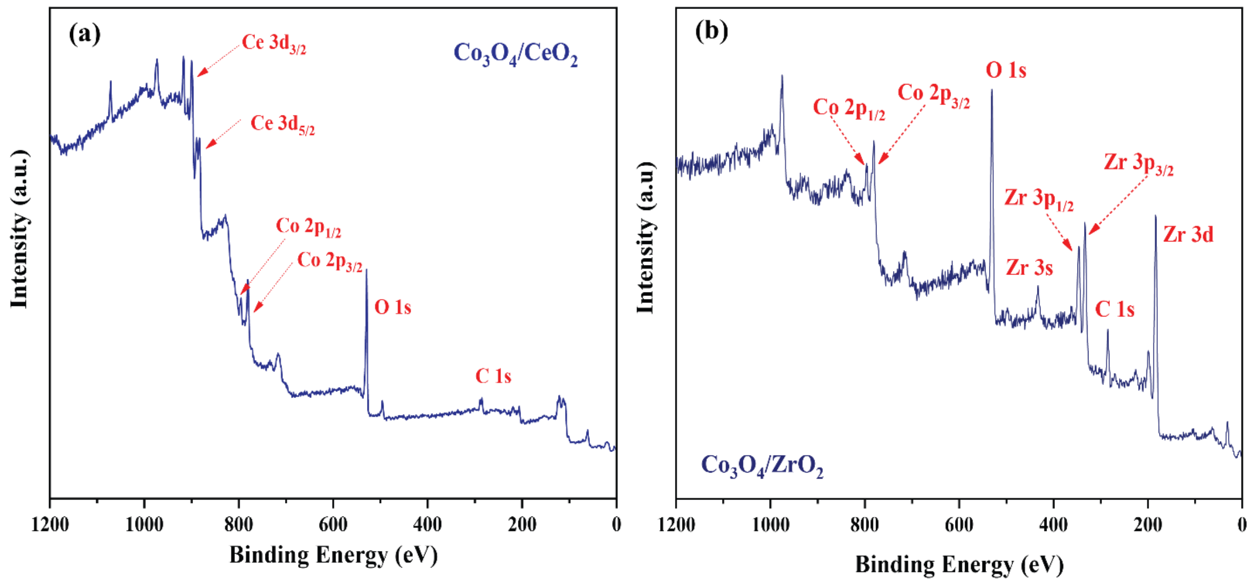

Figure S2: XPS survey scan of Co_3O_4/CeO_2 catalyst (b) XPS survey scan of Co_3O_4/ZrO_2

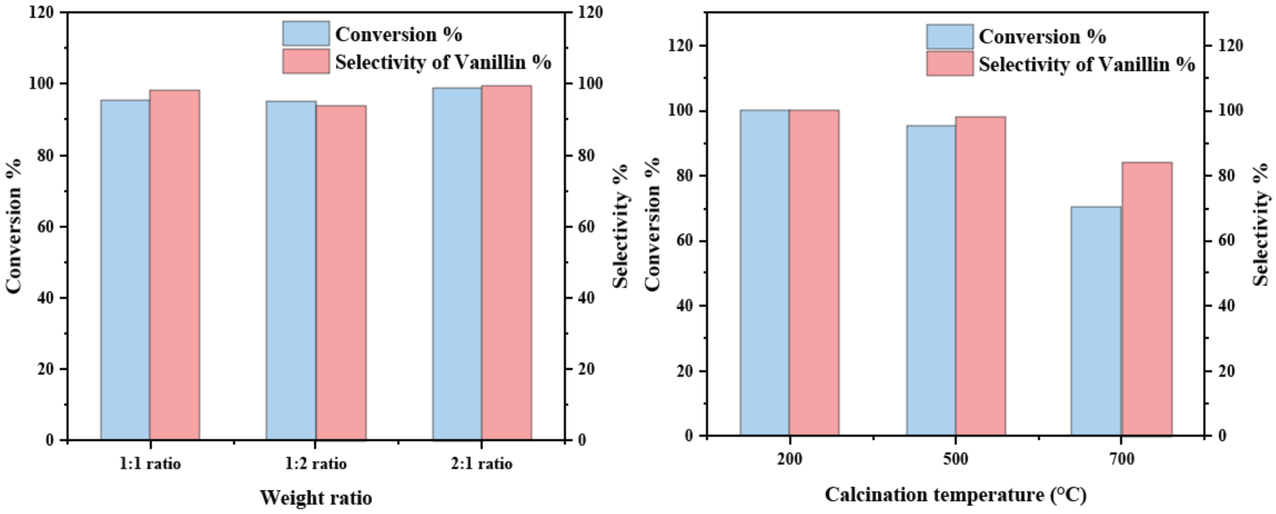

Figure S3: (a) The influence of metal composition on the vanillyl alcohol oxidation with different molar ratios of Co_3O_4 and CeO_2 in the composite at 70 °C for 24 hours. (b) The impact of the calcination temperature of the catalyst synthesis of Co_3O_4/CeO_2 for vanillyl alcohol oxidation at 70 °C for 24 hours

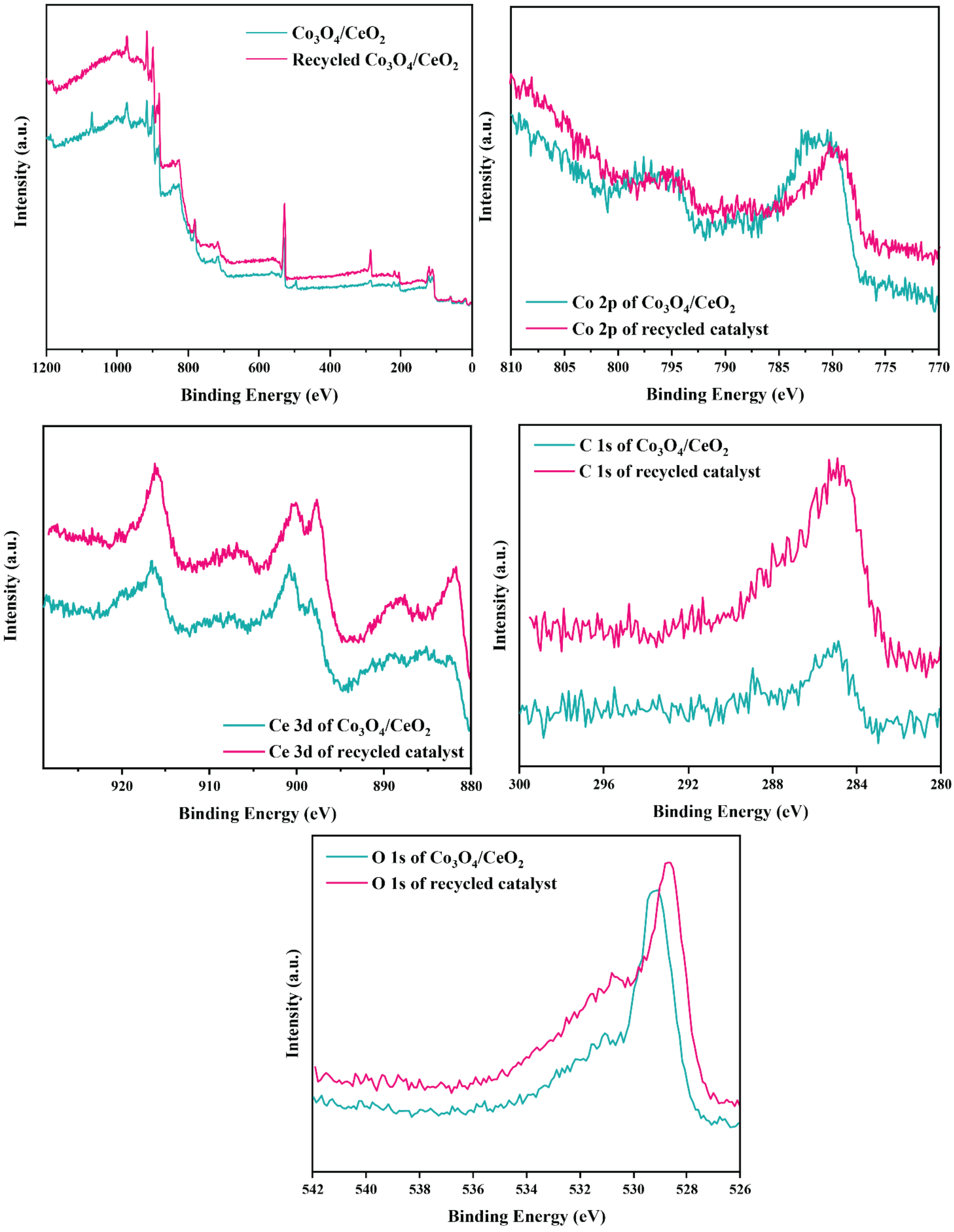

Figure S4: XPS analysis of the recycled catalysts

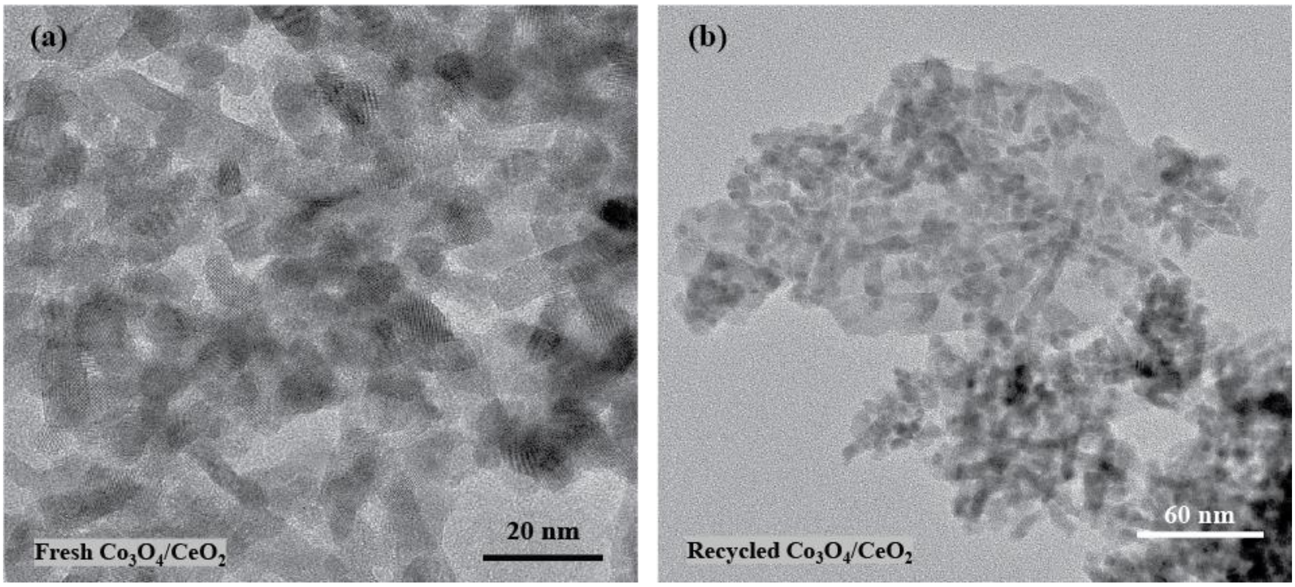

Figure S5: HR-TEM images of the (a) Fresh Co_3O_4/CeO_2 catalyst (b) Co_3O_4/CeO_2 after 4th reaction cycle

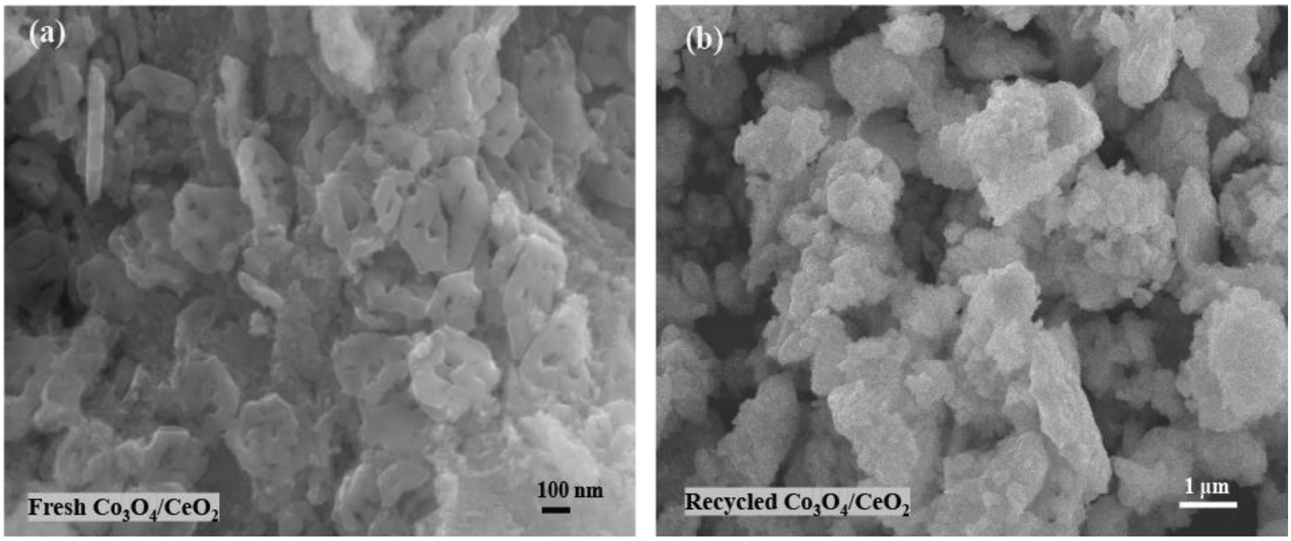

Figure S6: SEM images of the (a) Fresh Co_3O_4/CeO_2 catalyst (b) Co_3O_4/CeO_2 after 4th reaction cycle

Table S1: Adsorption amounts of vanillyl alcohol in acetonitrile onto catalyst supports at 70 °C

**Catalyst support**	**Adsorption amount (µmol/$g_{support}^{-1}$) of vanillyl alcohol**
Co_3O_4/CeO_2	58.38
Co_3O_4/ZrO_2	13.38

The adsorption was performed by adding a 10 mg catalyst into 3 ml of an aqueous solution containing 1 mmol adsorbate. The suspension was stirred for 30 minutes at 70 °C.

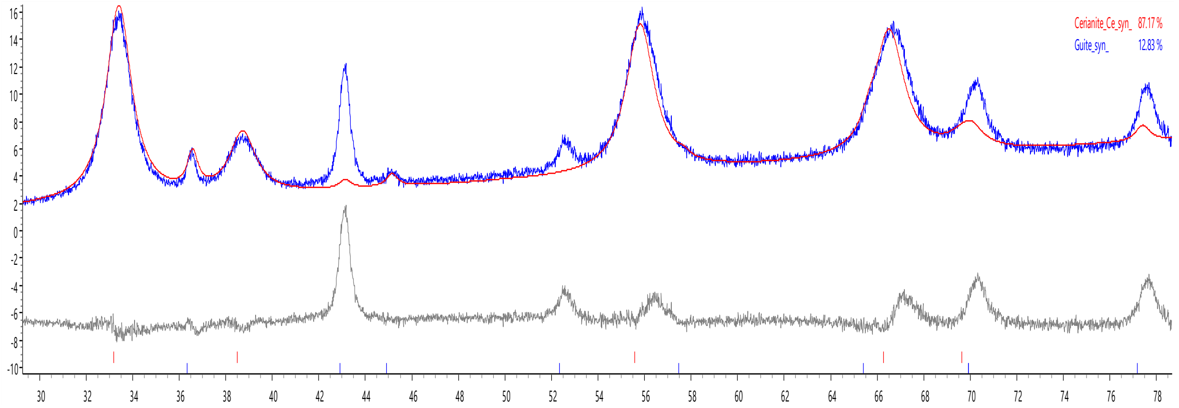

Figure S7: The Rietveld refinement pattern of Co_3O_4/CeO_2 composite

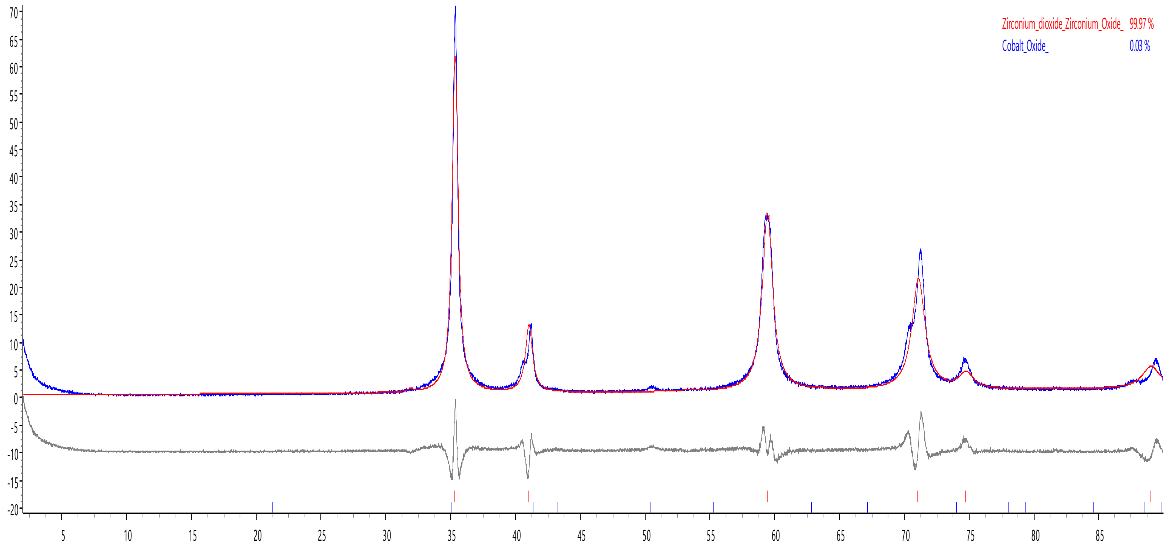

Figure S8: The Rietveld refinement pattern of Co_3O_4/ZrO_2 composite

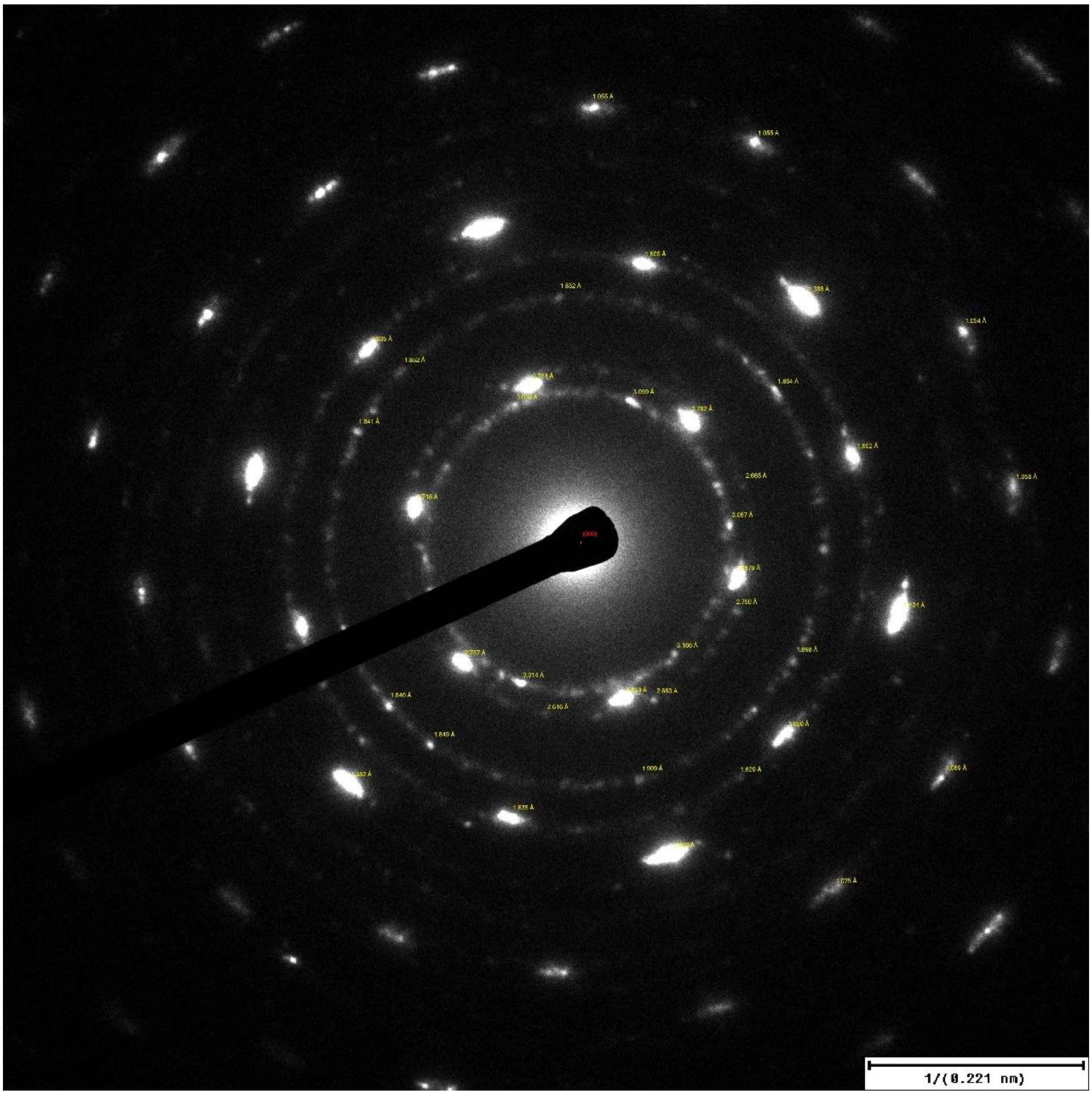

Figure S9: Annotated High-resolution image of the SAED pattern of Co_3O_4/CeO_2

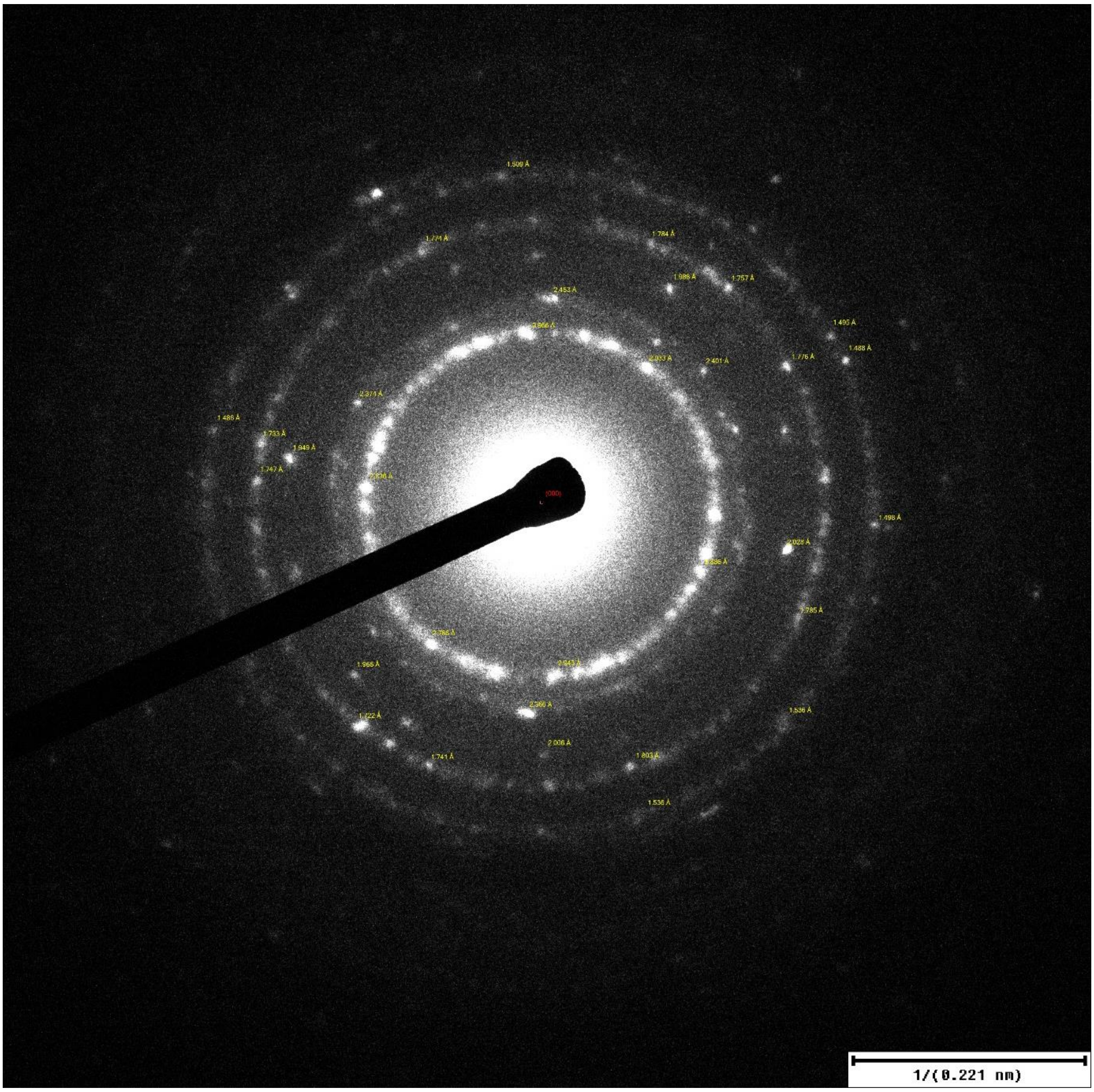

Figure S10: Annotated High-resolution image of the SAED pattern of Co_3O_4/ZrO_2
